# Gene Flow and Habitat Heterogeneity Shape Coexistence Dynamics of Arctic Charr Morphs in Connected Lakes

**DOI:** 10.1111/mec.70225

**Published:** 2026-01-16

**Authors:** Han Xiao, Arnar Pálsson, Zophonías O. Jónsson, Sigurður S. Snorrason

**Affiliations:** ^1^ Faculty of Life and Environmental Science University of Iceland Reykjavík Iceland

**Keywords:** coexistence, ddRADseq, geometric morphometrics, metapopulation, sympatric morph

## Abstract

Sympatric morphs provide valuable systems for studying incipient divergence despite incomplete reproductive isolation. In connected waterbodies with spatially heterogeneous habitats, one or more morphs may form metapopulation structures, generating eco‐evolutionary dynamics unlike those in single lakes. We studied the phenotypic and genome‐wide differentiation in Arctic charr ( *Salvelinus alpinus* ) in two Icelandic lakes: Thingvallavatn, known to harbour four distinct morphs, and a smaller downstream lake, Ulfljotsvatn. Our analyses confirm a single origin of charr polymorphism in this system, with all morphs present in both lakes. Relative morph abundances differ between the lakes: PL‐charr dominate in Thingvallavatn, whereas LB‐charr are most abundant in Ulfljotsvatn. Three morphs, large‐ (LB‐), small (SB‐) benthivorous and planktivorous (PL‐) charr, are genetically distinct. The system likely forms a source‐sink structure for both PL‐ and SB‐charr, though migration rates from Thingvallavatn vary remarkably, resulting in distinct population dynamics. Conversely, LB‐charr exhibit genetic differentiation between the lakes, suggesting the presence of a separate population in Ulfljotsvatn. While piscivorous (PI‐) charr appear genetically similar to PL‐charr, evidence suggests hybridisation between PI‐ and LB‐charr in both lakes. Moreover, the higher hybridisation in the downstream lake likely contributes to the observed erosion of genetic separation between LB‐ and PL‐charr in Ulfljotsvatn. These findings suggest that the complex interplay of habitat heterogeneity and morph‐specific migrations shapes the coexistence and eco‐evolutionary dynamics of sympatric charr morphs in the connected lakes. Our study highlights the importance of investigating early divergence in spatially complex systems to advance eco‐evolutionary research.

## 1 Introduction

Understanding how populations diversify in sympatry and new species form in the face of gene flow are two challenging questions in evolutionary biology. Efforts to tackle these questions often focus on different taxonomic levels or distinct eco‐evolutionary contexts (e.g., on within‐population polymorphism, Christie and Strauss 2019; and on well‐established species, Kautt, Machado‐Schiaffino, Torres‐Dowdall, and Meyer 2016), which may hinder a comprehensive view of evolutionary divergence as a multifaceted process. It has been proposed that sympatric morphs and young sister species can be arranged along a so‐called speciation continuum. This framework has been used to explore the emergence and maintenance of incipient biodiversity, emphasising the importance of tracking divergence with various strengths of reproductive isolation (Hendry 2009; Roux et al. 2016; Seehausen et al. 2014; Stankowski and Ravinet 2021).

### 1.1 Coexistence and Persistence of Sympatric Morphs

While within‐population divergence is frequently observed, sympatric speciation is rare, suggesting that ephemeral polymorphism is more common (Germain et al. 2021). There is substantial interest in inferring the evolutionary history of divergence initiation (e.g., Dean et al. 2019; Kautt, Machado‐Schiaffino, and Meyer 2016), however, the question of how sympatric morphs coexist with partial reproductive isolation and progress towards the completion of speciation is less explored. Newly evolved sympatric divergence is often driven by disruptive adaptation in heterogeneous environments, making it sensitive to environmental disturbance (Seehausen 2006). Oscillations in factors related to specific ecological opportunities can directly impact the survival of a specialised morph (Seehausen et al. 2008; Vonlanthen et al. 2012), and may also alter the interactions among coexisting morphs and other species. For example, they can reduce potential trophic niche partitioning, increase the risk of competitive exclusion (Germain et al. 2021), and the probability of hybridisation. Reproductive isolation between evolutionary young but distinct sympatric morphs is typically low to medium, likely maintained by extrinsic prezygotic barriers such as assortative mating (e.g., Elmer et al. 2009; Kroos et al. 2021; Podos 2010) and spatial/temporal segregation in mating preference (Hänniger et al. 2017; Walker et al. 1988), or extrinsic postzygotic barriers like reduced fitness of hybrids (Gow et al. 2007; Vamosi and Schluter 1999). In the absence of intrinsic postzygotic barriers, hybridisation between sympatric morphs can occur. If habitat changes lead to a sudden increase in hybrid fitness, or if premating isolation becomes ineffective (Gilman and Behm 2011; Seehausen et al. 2008), divergence may come to a halt or even collapse into hybrid swarms (Taylor et al. 2006).

These patterns and processes can become more complex in the context of metapopulation when one or more morphs have a patchy distribution. Unlike large and stable populations, a metapopulation consists of several local populations connected by migration. The extinction‐(re)colonisation dynamics among those interconnected populations can shape the evolution of a metapopulation differently (Hanski 1989; Levin 1995; Levins 1969). Both theoretical and empirical studies have substantially explored this subject in single‐species systems (Angst et al. 2022; Bonte and Bafort 2019; Freitas et al. 2022) and in metacommunities (Goodnight 2011; Holt 1997; Urban and Skelly 2006), but little attention has been directed towards sympatric polymorphism with incomplete reproductive isolation (but see Craze 2009; Manier and Arnold 2005). This may be partly due to the apparent scarcity of suitable systems, as only a few cases of sympatric morphs coexisting in connected, spatially heterogeneous habitats have been described (Doenz et al. 2019; Martin and Feinstein 2014; Salisbury et al. 2023). Although often overlooked, metapopulation structures of sympatric morphs may be more common than previously recognised, even if transient in nature. In systems such as connected lakes, patches can vary markedly in size, quality, and heterogeneity of habitats, leading to differences in ecological opportunity and carrying capacity to support each sympatric morph (Ebert et al. 2013). Moreover, morph‐specific traits (e.g., habitat preference and dispersal behaviours) and spatial configuration of patches (e.g., uninodal network between source and sink populations) may result in distinct migration patterns among localities and morphs (Barson et al. 2009; Lowe and McPeek 2014). Together, these factors can generate complex morph distributions and interactions across local populations within metapopulations, thereby providing valuable opportunities to investigate the eco‐evolutionary processes associated with morph divergence and reproductive isolation.

### 1.2 Sympatric Arctic Charr Morphs in Lake Thingvallavatn, Iceland

The salmonid Arctic charr ( *Salvelinus alpinus* ) is a cold‐water species widespread in arctic and subarctic waters with both anadromous and non‐anadromous populations (Klemetsen et al. 2003). Following the rapid retreat of the Icelandic icecap approximately 10,000–11,000 years ago, Arctic charr colonised accessible inland watersheds, and several lakes now harbour sympatric charr morphs (Brachmann et al. 2021; Gislason et al. 1999).

In Thingvallavatn, a large lake in southwest Iceland, four distinct charr morphs have been described based on head shape, coloration and size of adults (Malmquist et al. 1985), representing the highest intraspecific charr diversity in an Icelandic lake. The large and small benthivorous charr (hereafter referred to as LB‐ and SB‐charr, respectively) have a blunt snout and overshot mouth, and the planktivorous and piscivorous charr (PL‐ and PI‐charr) have a pointed snout and terminal mouth (Snorrason et al. 1989). These morphs also differ markedly in diet, habitat use and various life history characteristics, such as patterns of growth and sexual maturity (Jonsson et al. 1988; Sandlund et al. 1987). Studies using various types of genetic markers have shown that three of the four sympatric morphs, PL‐, LB‐ and SB‐charr, are genetically distinct, while the status of PI‐charr seems more complicated (Brachmann et al. 2021; Guðbrandsson et al. 2019; Kapralova et al. 2013, 2011; Magnusson and Ferguson 1987). Reconstructions of demographic history involving the three morphs (LB‐, SB‐ and PL‐charr) support a scenario with a period of divergence in allopatry followed by secondary contact (Brachmann et al. 2022; Kapralova et al. 2011). The rapid evolution of sympatric charr morphs in Thingvallavatn is thought to have been driven by disruptive selection associated with differential resource use, likely facilitated by the lake's large size (83 km^2^) and high habitat heterogeneity (Brachmann et al. 2021; Smith and Skúlason 1996).

### 1.3 Potential Metapopulation Dynamics of Sympatric Charr Morphs in Connected Lakes

Although Thingvallavatn is the headwater for the whole drainage, it is not completely isolated from downstream water bodies. A short river (Efra Sog, ~1.1 km) flows rapidly from it into Ulfljotsvatn, a much smaller (3.6 km^2^) and shallower lake lying approximately 21 m lower in elevation. Below Ulfljotsvatn, two large waterfalls prevent the upward migration of fish from further down in the system (Jónasson 1992).

Charr polymorphism has also been documented in Ulfljotsvatn (Woods et al. 2012), although detailed information on the morphology and life history traits of fish in the lake has been lacking. Annual fishery reports in the period of 1992–2000 indicated that all four Thingvallavatn morphs were also found in Ulfljotsvatn, but morph classification was challenging (Jóhannsson and Jónsson 2000). In most years, the proportions of morphs were similar, with LB‐charr being the most abundant while SB‐charr was rare. The evolutionary relationship among charr morphs in the Thingvallavatn‐Ulfljotsvatn system remains unknown but may be tightly dependent on genetic connectivity between the two lakes. Upstream migration of fish from Ulfljotsvatn to Thingvallavatn was likely feasible until 1959 when a dam was constructed at the outlet of Thingvallavatn for the hydroelectric power station, Steingrimsstod (Jónasson 1992). Since then, only downstream migration has been possible, either through a bottom sluice gate at the dam or through the turbines of the power plant.

Although occurrences of sympatric morphs derived from a single origin across localities appear rare in salmonids (Salisbury and Ruzzante 2022), the independent evolution of similar charr polymorphism in each lake is improbable. Given the extensive differences in physical characteristics and habitat availability between the two lakes, Thingvallavatn and Ulfljotsvatn may form a source‐sink system harbouring metapopulations of one or more charr morphs. And thus, the persistence of any charr morph in Ulfljotsvatn is mainly a function of immigration from Thingvallavatn and habitat quality. In the extreme scenario where corresponding ecological opportunities are insufficient to sustain a given morph, all observed individuals of that morph may originate from Thingvallavatn via immigration. Conversely, if a morph can successfully survive and reproduce in Ulfljotsvatn, local adaptation and divergence are likely to occur in the absence of continued downstream gene flow.

To better understand the eco‐evolutionary dynamics of charr morphs in the Thingvallavatn‐Ulfljotsvatn system, particularly the mechanisms facilitating their coexistence in Ulfljotsvatn, we examined both the phenotypic and genomic variation in sympatric charr morphs in these two connected lakes using geometric morphometrics, diet analysis, genome‐wide SNPs acquired via double digest Restriction‐site Associated DNA sequencing (ddRADseq) and two morph‐associated mitochondrial markers. We hypothesise that the charr polymorphism in the two lakes evolved once, with similar morphs from each lake sharing the closest ancestry. However, the persistence of different morphs in the downstream lake may vary due to differences in immigration rates and resource availability for each morph. Additionally, reduced ecological opportunities in Ulfljotsvatn may have altered interactions among morphs, particularly increasing hybridisation, which can result in reduced genetic differentiation among some morphs. The findings illuminate how sympatric morphs with incomplete reproductive isolation coexist in connected lakes that differ in key environmental characteristics and provide insight into the unique roles of metapopulation structures in shaping adaptive divergence.

## 2 Materials and Methods

### 2.1 Study System

Thingvallavatn and Ulfljotsvatn drain via river Sog and join with the glacial river Hvita to form river Olfusa. Thingvallavatn has a surface area of 83 km^2^, mean depth of 34 m, maximum depth of 114 m, average discharge rate of 100 m^3^ per second and retention time of ~330 days (Adalsteinsson et al. 1992). Being a deep subarctic oligotrophic lake, the principal habitats for the fish of Thingvallavatn consist of an extensive pelagic zone with a relatively simple community of zooplankton (Antonsson 1992) and spatially complex benthic zones, the shallow parts (depth of 0–8 m) with uneroded lava or lava rubble and dominated by diatoms (G. St. Jónsson 1992), below which the bottom becomes muddy with stands of *Nitella apaca* algae that may extend down to 20 m depth (Kairesalo et al. 1992). Both habitats support a diverse invertebrate community (Lindegaard 1992). Ulfljotsvatn is a much smaller and shallower lake, with a surface area of 3.6 km^2^, a mean depth of 4.7 m, a maximum depth of 34.5 m and a retention time of only 1 day (Aðalsteinsson et al. 1989). The shallow littoral zone (0–1.5 m) consists of stones of various sizes and some sandy stretches. Lava substrates are limited to small areas (less than 5% of the shoreline) in the north of Ulfljotsvatn. Deeper areas (mostly at 1.5–5 m depth) are covered with sand or mud. The Thingvallavatn‐Ulfljotsvatn system harbours two other fish species, the threespine stickleback ( *Gasterosteus aculeatus* ) and brown trout ( *Salmo trutta* ).

### 2.2 Fish Sampling and Morph Assignment

**FIGURE 1:**
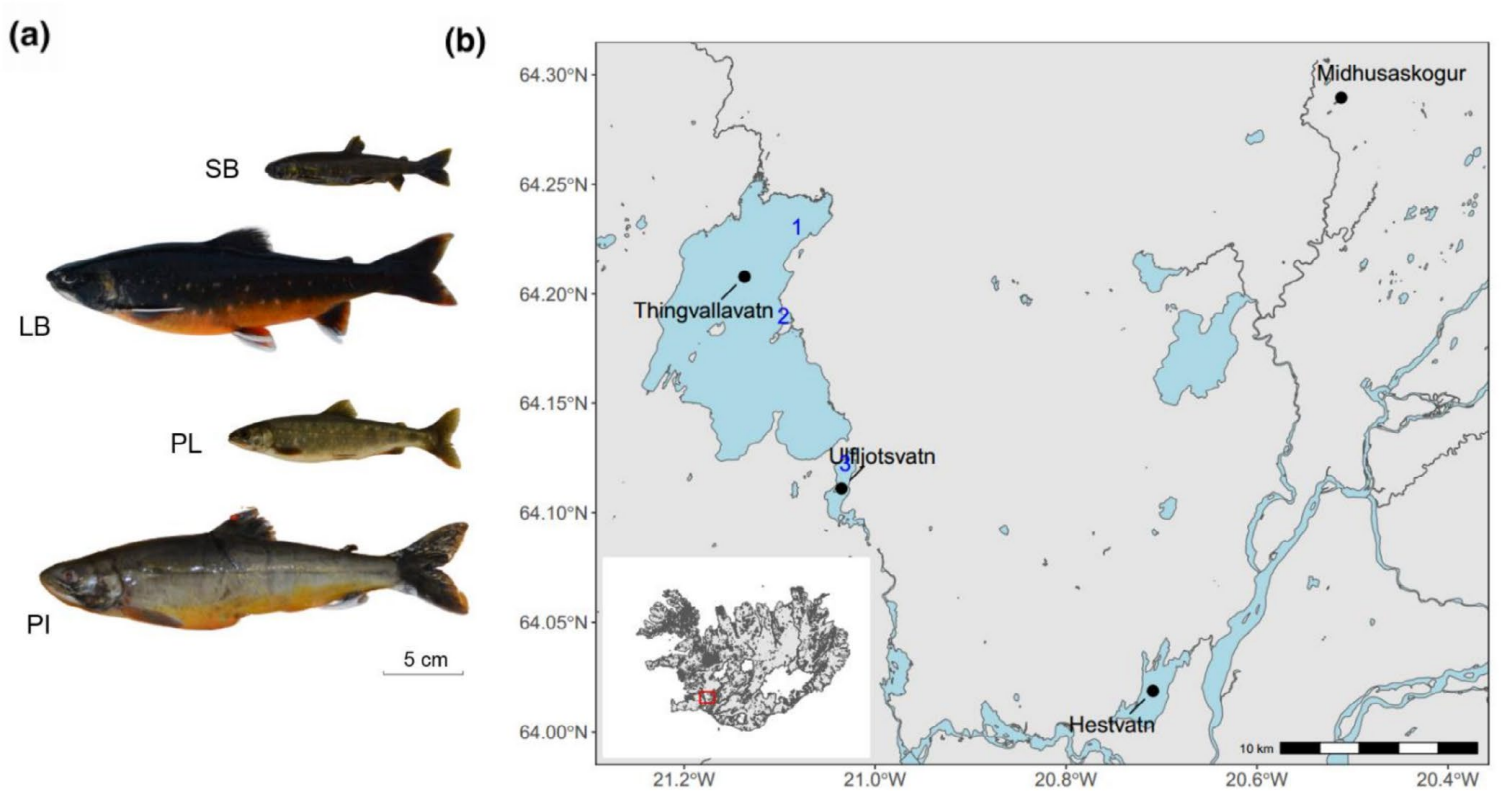
The four sympatric Arctic charr morphs in Thingvallavatn and the study system. (a) From top: Small benthivorous (SB), Large benthivorous (LB), Planktivorous (PL), and Piscivorous (PI) charr. (b) Map shows the sampling locations including two reference populations and sampling sites in Thingvallavatn and Ulfljotsvatn: 1. Olafsdrattur 2. Mjoanes peninsula 3. Northeast shore of Ulfljotsvatn.

Fish were collected from Thingvallavatn between 2015 and 2018, and from Ulfljotsvatn in 2015 and 2017. In Thingvallavatn, gill nets, 30 m long and 1.8 m high, with different mesh sizes (10–60 mm knot to knot) were used. Nets were laid overnight at different depth ranges (2–20 m) and in different months to catch all morphs at the peak of their spawning (Skúlason et al. 1989). In Thingvallavatn, the sampling sites were Olafsdrattur, a known spawning site of LB‐charr in August, and at Mjoanes, where PL‐, PI‐ and SB‐charr are found with ripe gonads in September and October. In Ulfljotsvatn, where spawning grounds and spawning time (if any) of charr are unknown, fish were sampled during September and October using gill nets deployed at the north‐eastern shore at depths from 1.5 to 4 m. To analyse the phylogenetic relationships of charr in the lake system, we also obtained reference samples from two populations: a PL‐like form from Hestvatn (collected by gill net in 2012, *N* = 32) and a benthic dwarf form from Midhusaskogur (collected by electrofishing in 2015, *N* = 11) (Figure 1b).

For all lakes, each fish was photographed from the left side using a Nikon digital single‐lens reflex camera (NIKON D3200), and a biopsy of a fin clip or muscle tissue was collected and preserved in 96% ethanol. Additionally, gender and sexual maturity stage were determined. Stomach contents of all Ulfljotsvatn charr were analysed in the laboratory by spreading on a Petri dish and identifying key taxa or species when possible.

Morph assignment of fish from Thingvallavatn was conducted in the field based on visual inspection of head morphology and body coloration (Snorrason and Skúlason 2004). Phenotypically intermediate fish (if any) were excluded, and only sexually mature fish were used in further analyses. In Ulfljotsvatn, morph assignment was first performed in the field during sampling, followed by a second round based on photographs in the laboratory. Only specimens assigned to the same morph by both assessments were included as definite morphs. Fish that could not be assigned to any morph category were classified as ‘unassigned’ for subsequent analyses. Additionally, visually identified PL‐individuals found with snails in their stomachs were defined as ‘unassigned’, and only LB‐charr over 20 cm were retained to minimise potential allometric effects on the morphometric analysis.

### 2.3 Shape Analysis

Landmark‐based geometric morphometric approaches were used to quantify head shape characteristics (Adams et al. 2004; James Rohlf and Marcus 1993). To extract shape information, a set of 18 landmarks and semi‐landmarks (Figure S1) were digitised by the same investigator on photographs of each individual's head using tpsDig2, downloaded from the Stony Brook morphometric website (https://www.sbmorphometrics.org/). To remove variations (position, orientation and scale) irrelevant to shape, a Generalised Procrustes Analysis (GPA) was performed with the R package *geomorph* v. 4.0.1 (Baken et al. 2021).

To study head shape variation among sympatric morphs within and between lakes, we first performed a principal component analysis (PCA) to graphically evaluate variations along the first two principal components (PCs) across all categories, including unassigned samples from Ulfljotsvatn. For a subset of specimens, excluding unassigned samples, we conducted a series of Procrustes MANOVA tests using *geomorph* (procD.lm function) to investigate associations between variables (morph, lake and unique morph) and shape (Procrustes coordinates), including all possible interactions. We defined unique morphs as combinations of morph and lake (e.g., ThSB and UlfSB as two unique morphs). Lastly, we tested for allometry (the covariation between size and shape) using log‐transformed centroid size with morph, morph and lake, and unique morph as variables. All possible interactions were included. Differences in allometry among unique morphs were visualised using the plotAllometry function in *geomorph*.

### 2.4 ddRADseq and KASP Genotyping

We used a standard phenol/chloroform protocol to extract DNA from fin clips or muscle tissue of collected samples. DNA concentration was measured on a NanoDrop spectrophotometer (Thermo Scientific), and DNA quality was assessed using gel electrophoresis on a 0.8% agarose gel.

#### 2.4.1 ddRAD Library Preparation and Sequencing

Libraries for ddRADseq were prepared according to the previously described method (Peterson et al. 2012) with some modifications. A detailed explanation can be found in Lagunas et al. (2023). Briefly, genomic DNA was equalised to a concentration of 30 or 50 ng/μL and digested with ApeKI and BamHI HF (New England Biolabs Inc.). To minimise potential library effects, 96 samples from at least four distinct types (either unique morphs or reference populations) were pooled into one library after ligation with individually unique combinations of *Ape*KI adapters (4 bp barcodes) and *Bam*HI HF adapters (6 bp barcodes). Fragments between 360 and 440 bp were then selected using Pippin prep (Sage Science, Beverly, MA, USA) and amplified via PCR. After final purification with magnetic beads (Macherey‐Nagel, Düren, Germany), the concentration of each library was measured using a Qubit 3.0 Fluorometer with the dsDNA BR Assay (Life Technologies, Carlsbad, CA, USA). The fragment size distribution in the final library was first checked by gel electrophoresis on a 2% Agarose gel and then on an Agilent 2100 Bioanalyzer High Sensitivity Chip (Agilent Technologies Inc.). A subset of the libraries was sequenced on Illumina MiSeq for quality control to confirm the success of library preparation. Finally, each library was sequenced on a single lane of Illumina HiSeq X‐Ten (2 × 150 paired‐end reads) at BGI Tech Solutions (Hong Kong).

#### 2.4.2 KASP Genotyping

We genotyped two mitochondrial polymorphic markers previously described in Thingvallavatn morphs (Gudbrandsson et al. 2016) using a Kompetitive Allele‐Specific PCR (KASP) assay. The 12S rRNA polymorphism (m1829G > A) was found to be enriched in SB‐charr but also detected at lower frequency in LB‐charr, while m3211T > C in the 16S rRNA gene appeared specific to LB‐charr and occurred at high frequency. We genotyped the same samples as for the ddRADseq except for the unassigned fish. Primers were designed based on sequences 102 bp upstream and 102 bp downstream of each marker (Table S1). KASP reactions were conducted following a 61°C–55°C touchdown protocol (LGC Genomics, USA) and assembled in a 96‐well plate with 5 μL of 2× KASP Master mix, 0.14 μL of KASP Assay mix and either 5 μL of genomic DNA (50 ng/μL) or water as control. Up to 95 samples and a water control were processed in QuantStudio 3 Real‐Time PCR System equipment from Applied Biosystems instruments. Result visualisation and genotype identification were conducted in the Thermo Fisher cloud software (Thermo Fisher Scientific Inc.).

### 2.5 ddRADseq Genotyping and Bioinformatics

Raw sequence data quality was assessed with FastQC version 0.11.7 (Babraham Bioinformatics, Babraham Institute). The process_radtags pipeline in Stacks version 2.41 (Rochette et al. 2019) was used for demultiplexing, barcode removal and trimming raw sequence data to 115 bp. Processed paired‐end reads for each specimen were aligned to the *Salvelinus* sp. Genome (https://www.ncbi.nlm.nih.gov/datasets/genome/GCF_002910315.2/) using global alignment with *BWA‐MEM* (H. Li 2013). We then ran the gstacks pipeline in Stacks with the option ‐‐min‐mapq 20 to build RAD loci and identify SNPs. SNP calling followed a similar approach to (Cerca et al. 2021), with each unique morph classified as a distinct population for the filtering. Briefly, the populations pipeline in Stacks was first run with all sequenced individuals requiring a call rate for each SNP greater than 70% across all samples (‐P 7) and 66% within each unique morph (−r 0.66). After obtaining the initial dataset, mean depth per individual, individual missingness and inbreeding coefficient (*F*) were calculated using VCFtools version 0.1.15 (Danecek et al. 2011). Individuals with a mean coverage lower than 10 or missingness higher than 30% (except for the Midhusaskogur reference population as 45%) were excluded in downstream filtering. Pairwise genome similarity was calculated using the R package *radiator* (Gosselin et al. 2020). If duplicated samples were detected, only one was retained for analysis. After poorly sequenced samples were identified and removed, we generated two datasets: a ‘global’ dataset containing charr morphs from the Thingvallavatn‐Ulfljotsvatn system and reference populations, and a ‘regional’ dataset with only charr from the two connected lakes. The minimum number of populations required to process a locus was set to be 7 (−p 7) for the ‘global’ dataset and 6 (−p 6) for the ‘regional’ dataset. For both datasets, we applied the following criteria: (1) the minimum minor allele count to be 3 (−‐min‐mac 3); (2) the maximum observed heterozygosity to be 0.60 (−‐max‐obs‐het 0.60); (3) the minimum percentage of individuals in a population required to process a locus for that population to be 66% (−r 0.66) and (4) only the first SNP of each locus was kept (−‐write‐single‐snp) for further analyses. The export was ordered (−‐ordered‐export) along the reference genome and outputs in different formats were generated or converted using Stacks and Plink version 1.9 (Chang et al. 2015).

### 2.6 Broad Connectivity and Phylogenetic Reconstruction

The phylogenetic relationship of charr from these two lakes and nearby reference populations was explored using the ‘global’ dataset in two ways. First, we used a model‐free method of principal component analyses (PCAs) with the R package *adegenet* (Jombart and Ahmed 2011). Missing data were accounted for using the default settings by replacing them with mean allele frequency values. Second, a maximum‐likelihood tree for all individuals was estimated using RAxML‐ng version 1.0.3 (Kozlov et al. 2019). We performed 20 tree searches using 10 random and 10 parsimony‐based starting trees under the GTR + GAMMA model with default parameters. The phylogenetic tree with the lowest final log likelihood was selected and visualised using Interactive Tree of Life (iTOL) v6 (Letunic and Bork 2024).

### 2.7 Population Genomics

To understand the genomic relationships among sympatric charr morphs within the Thingvallavatn‐Ulfljotsvatn system, we conducted a series of analyses using the ‘regional’ dataset unless otherwise stated. Genome‐wide genetic diversity statistics, including average individual observed heterozygosity (*H*_O_) and expected heterozygosity (*H*_E_) were calculated for each unique morph using VCFtools, and Weir and Cockerham's pairwise *F*_ST_ (Weir and Cockerham 1984) was calculated using the populations pipeline.

#### 2.7.1 Population Structure

Population structure among sympatric morphs in the lake system was first explored with the model‐free approach of PCA using the R package *adegenet*, and fish from both lakes were analysed together. Using the model‐based approach of ADMIXTURE version 1.23 (Alexander et al. 2009), we then performed analyses on each lake separately with predefined clusters (*K*) ranging from 1 to 4, followed by the analysis of fish from both lakes together with *K* ranging from 1 to 8. The most likely numbers of clusters were detected based on a tenfold cross‐validation technique using *structureselector* (Y. L. Li and Liu 2018). Due to uneven sampling size and potential impact of including unassigned fish from Ulfljotsvatn as an artificial group, four additional methods (MedMedK, MedMeanK, MaxMedK, and MaxMeanK) were also applied for best supported cluster number estimation (Puechmaille 2016). The output of 10 independent runs across each *K* was averaged and visually displayed using Clumpak with default advanced options (Kopelman et al. 2015). Lastly, we built individual‐based phylogenetic networks of morphs from both lakes with SplitsTree version 4.17.1 (Huson and Bryant 2006) using the NeighborNet method based on uncorrected P distances.

#### 2.7.2 Tests for Gene Flow

To explore the effects of migrations among unique morphs within the Thingvallavatn‐Ulfljotsvatn system, we tested potential admixtures and gene flow using TreeMix version 1.13 (Pickrell and Pritchard 2012) and BA3‐SNP (Mussmann et al. 2019). SB‐charr from Ulfljotsvatn were excluded due to the minute sample size (*N* = 4). We performed the analyses separately for morphs within each lake and then combined morphs from both lakes to compare the results. Additionally, we used the program Dsuite (Malinsky et al. 2021) to statistically test for introgression by examining allele sharing among unique morphs under an ABBA‐BABA framework.

For the TreeMix analyses, maximum likelihood phylogenetic trees were first built for reference populations and morphs from the lake system using allele frequency data extracted from the ‘global’ dataset. We rooted the trees with Hestvatn, and up to 8 migration events (M) for Thingvallavatn, 6 migration events for Ulfljotsvatn and 11 migration events for both lakes combined were fitted onto the corresponding trees. Ten iterations at each value of *M* were performed using random subsets of 80% of the total SNPs for each case, and the optimal number of migration edges for each corresponding analysis was determined using the R package *optM* (Fitak 2021). The results were visualised in R version 4.1.1. For the BA3‐SNP analysis, we first ran BA3‐SNPs‐autotune on each dataset to find optimal mixing parameters for each run, with a maximum of 15 exploratory analyses employing 10,000 MCMC generations. Then BA3‐SNPs was run with the final mixing parameters for 10 million iterations, discarding the first 1 million iterations on each corresponding case.

Lastly, we calculated Patterson's D statistics (Durand et al. 2011; Green et al. 2010), corresponding Z‐scores and associated *p*‐values, as well as the f_4_ ratio (Patterson et al. 2012) for every possible (((P1, P2), P3), OG) topology of charr morphs/populations in the system, using the Dtrios module from Dsuite with default settings. Hestvatn was specified as the outgroup and the combined‐lake phylogenetic tree constructed in TreeMix was supplied to the Fbranch module to calculate the f‐branch statistic, which helps interpret correlated f_4_‐ratio results and assign gene flow to specific branches on a phylogeny. We focused primarily on validating migration events detected by TreeMix while treating other introgression signals, especially the ones between different morphs across lakes, with caution, as these may not reflect real biological processes but rather result from the complicated relationships among morphs and the relatively low accuracy based on RADSeq data (Malinsky et al. 2021).

#### 2.7.3 Genetic Differentiation and Persistence of Sympatric Morphs by Lake

To investigate the extent and consequences of hybridisation between morphs in the system, we focused on LB‐ and PL‐charr, which are genetically distinct and abundant in both lakes. First, Weir and Cockerham's *F*_ST_ was computed for each SNP between PL‐ and LB‐charr in each lake using VCFtools. Next, the genome‐wide distribution of *F*_ST_ for each lake was plotted and compared. Finally, SNPs with an *F*_ST_ greater than 0.5 in both lakes were extracted, and their functional annotations were catalogued using the *Salvelinus* sp. Genome on NCBI and GeneCards (Stelzer et al. 2016).

## 3 Results

### 3.1 Morph Identification and Assignment

In Thingvallavatn, all fish were successfully assigned to morphs. In contrast, around one third (74 out of 232, 31.9%) of the charr caught in Ulfljotsvatn could not be assigned to a specific morph. For two‐thirds of these unassigned fish (49 out of 74, 66.2%), we were unable to distinguish between benthic and limnetic form. For the assigned fish, the most abundant morph was LB‐charr (84, 36.21%), followed by PL‐charr (42, 18.10%) and PI‐charr (28, 12.07%). Only four specimens were classified as SB‐charr across the two sampling years, three of which were sexually mature. The diets differed among the four morphs in Ulfljotsvatn (Table S2). LB‐charr primarily consumed snails, followed by insect remains and benthic crustacea. PL‐charr, by contrast, mainly fed on benthic crustacea, chironomid larvae, and insects. Most PI‐charr had an empty stomach, and notably, none had eaten fish. Interestingly, some PI‐ and SB‐charr had ingested salmonid eggs. Five individuals initially classified as PL‐charr, which had consumed snails, were subsequently reclassified as unassigned fish. A summary of the samples used for further analyses is presented in Table 1.

**TABLE 1:** Sympatric Arctic charr morphs in the Thingvallavatn‐Ulfljotsvatn system included in morphological and genetic analyses with corresponding sample size (*n*), average individual observed heterozygosity (*H*_O_) and expected heterozygosity (*H*_E_).

Lake	Morph	Code	Morphology (*n*)	Genetics^a^ (*n*)	*H*_O_	*H*_E_
Thingvallavatn	Large benthivorous	ThLB	33	37	0.149	0.191
	Small benthivorous	ThSB	49	31	0.158	0.187
	Planktivorous	ThPL	55	32	0.168	0.189
	Piscivorous	ThPI	25	22	0.170	0.190
Ulfljotsvatn	Large benthivorous	UlfLB	35	34	0.168	0.188
	Small benthivorous	UlfSB	4	4	0.172	0.186
	Planktivorous	UlfPL	21	19	0.166	0.190
	Piscivorous	UlfPI	24	25	0.179	0.191
	Unassigned	UlfUN	39	39	0.178	0.191

^a^ Final number after individuals with poor sequencing were removed.

### 3.2 Shape Analysis

**FIGURE 2:**
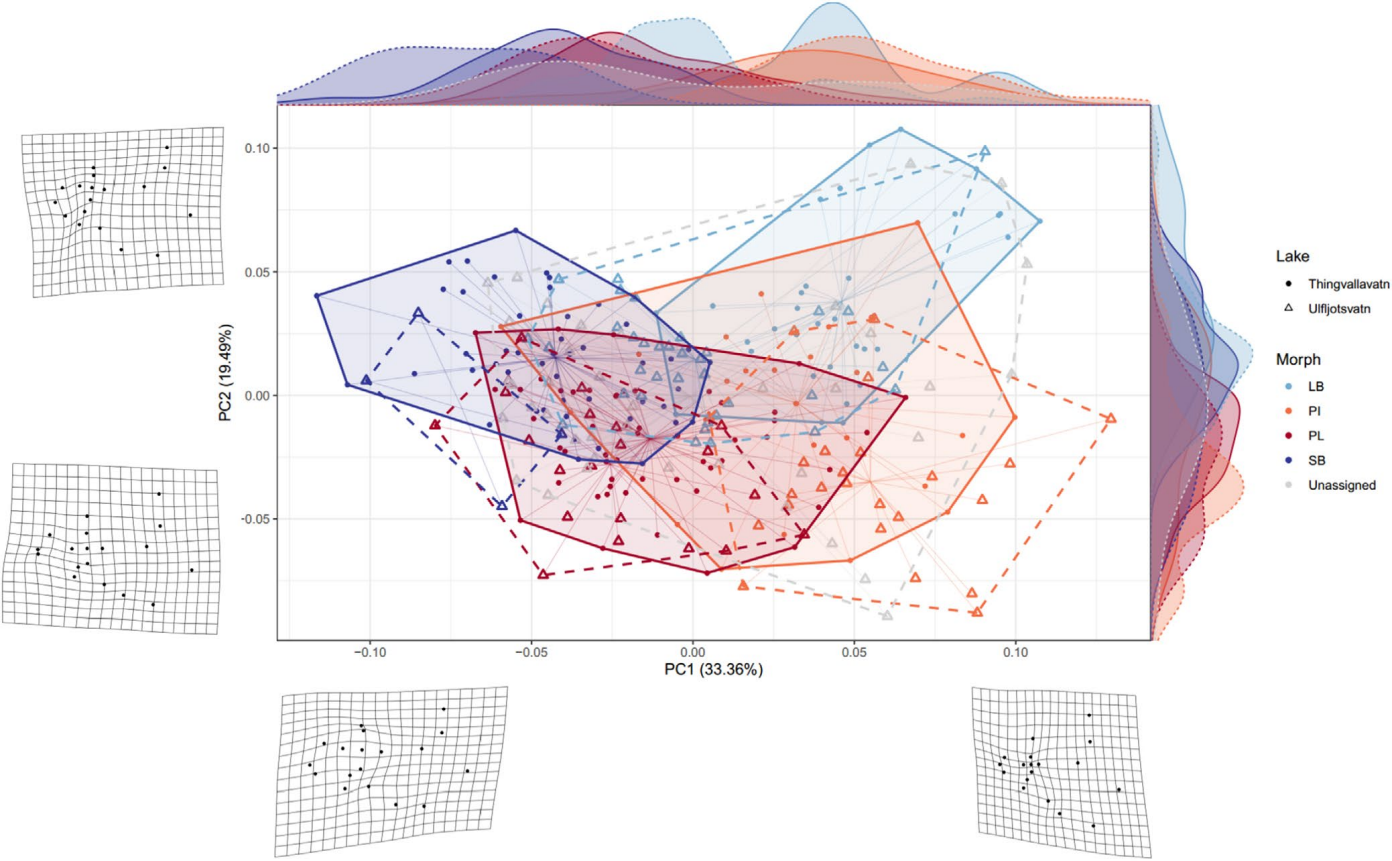
Variation in head shape among Arctic charr morphs in Thingvallavatn and Ulfljotsvatn represented along the first two principal components (PCs) of a PCA. Density plots along each PC are displayed on the margins. Points represent individual fish, while polygons indicate the convex hulls for each unique morph, with individuals connected to their corresponding centroids. Colours correspond to different morph and symbol shapes denote lakes (see legend). Solid lines represent Thingvallavatn, and broken lines represent Ulfljotsvatn. Deformation grids along each axis depict the changes in head shape associated with maximum and minimum PC values (no warp magnification).

Morphological analysis revealed significant differences in head shape among sympatric morphs within each lake, as indicated by a principal components analysis (PCA) on Procrustes shape coordinates without a priori grouping (Figure 2). PC1 captured 33.3% of the variance and distinguished small morphs (SB‐ and PL‐charr) from the large morphs (LB‐ and PI‐charr). The shape variation along PC1 mainly reflected differences in head depth, the proportional size of eyes, and the maxilla. PC2 explained 19.5% of the variance and separated morphs along the benthic–limnetic axis. Shape changes along PC2 were predominantly driven by the relative length of lower jaw, location of eyes and maxilla shape. Specifically, benthic morphs exhibited a subterminal mouth with a shorter and rounder maxilla, whereas limnetic morphs had terminal mouths with longer and narrower maxillae. Unassigned specimens from Ulfljotsvatn were dispersed throughout the tangent space. Corresponding morphs from the two lakes exhibited considerable overlap along the first two PC axes of variation, with the degree of overlap differing among morphs. PL‐charr was the least distinguishable between lakes, while the large morphs showed greater separation.

Procrustes ANOVA revealed that head shape was significantly associated with morph, lake, and their interaction, explaining 30.00%, 2.08% and 4.87% of the shape variation, respectively (Table S3). Next, we used unique morph as an ecomorphological factor, which explained 36.94% of the variation in head shape. We also detected a notable relationship between log‐transformed size and head shape, which accounted for 22.67% of the variation (Table S4). Morph, lake, and all two‐way interactions also significantly influenced head allometry, suggesting that at least some unique morphs exhibited distinct allometric patterns. When morph and lake were combined as unique morph in the model, this factor explained 17.78% of the shape variation. Plotting log transformed centroid size against PC1 scores for shape revealed that larger unique morphs experienced steeper changes in head shape with increasing size than smaller unique morphs (Figure S2).

### 3.3 Sequencing and Genotyping Summary

Using ddRADseq, we obtained an average of 7.1 ± 5.0 (mean ± SD) million mapped reads per individual. Twelve individuals were excluded after initial filtering due to low coverage. Following stringent filtering, 11,160 and 7840 SNPs were retained for the ‘global’ and ‘regional’ dataset, respectively (Table S5). For morph‐specific mitochondrial polymorphism detection, marker m1829 failed to amplify in five individuals and marker m3211 failed to amplify in six individuals. Three individuals could not be successfully genotyped for either marker.

### 3.4 Broad Connectivity and Phylogenetic Reconstruction

As expected based on the geography of the Olfusa catchment, charr from the Thingvallavatn‐Ulfljotsvatn system formed a distinct genetic cluster, clearly separated from the Hestvatn and Midhusaskogur populations by the first two PCs (Figure S3a). Further PCs did not distinguish between lakes or morphs within Thingvallavatn and Ulfljotsvatn. The maximum‐likelihood tree similarly supported the shared ancestry of charr within the system, clearly separate from the two reference populations (Figure S3b).

### 3.5 KASP Genotyping

We genotyped two mitochondrial markers to test separation of maternal lineages. These markers formed three haplotypes, two of which were unique to the Thingvallavatn‐Ulfljotsvatn system (Figure S4). Haplotype TG, representing the ancestral state, was found in all individuals from reference populations and was shared across all morphs in the two lakes. Haplotype CG was frequent (84.8%) as expected in LB‐charr from Thingvallavatn but occurred at a surprisingly low frequency (12.1%) in LB‐charr from Ulfljotsvatn. Haplotype TA was predominantly found in SB‐charr in both lakes, with a frequency of 58.1% in Thingvallavatn and 25% in Ulfljotsvatn respectively. However, neither of these two haplotypes was exclusive to LB‐ or SB‐charr, as they were detected in one or more other charr morphs across the lakes.

### 3.6 Basic Statistics and Population Structure of Charr Morphs in the Connected Lakes

**FIGURE 3:**
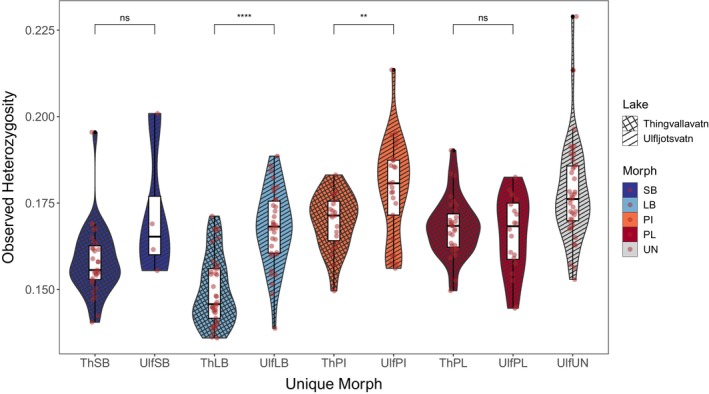
Observed heterozygosity (*H*_O_) per individual, for each unique morph in the Thingvallavatn‐Ulfljotsvatn system. Data are represented as boxplots with superimposed density distributions (coloured by morph and patterned by lake: Hatched for Thingvallavatn and lined for Ulfljotsvatn), and individual specimens (red circles). Pairwise comparisons of mean *H*o between morphs across lakes were done with Wilcoxon test (ns: *p* > 0.05, **p < 0.01, ****p < 0.0001).

Individual mean heterozygosity varied by morphs within each lake, with observed heterozygosity (*H*_O_) consistently lower than expected heterozygosity (*H*_E_) across all morphs (Table 1). The highest *H*_O_ was observed in PI‐charr in both lakes. Specifically, *H*_O_ in LB‐ and PI‐charr was significantly higher in Ulfljotsvatn than in Thingvallavatn, while no difference was found in PL‐ and SB‐charr across the two lakes (Figure 3). Unassigned fish in Ulfljotsvatn had the broadest distribution of *H*_O_.

**FIGURE 4:**
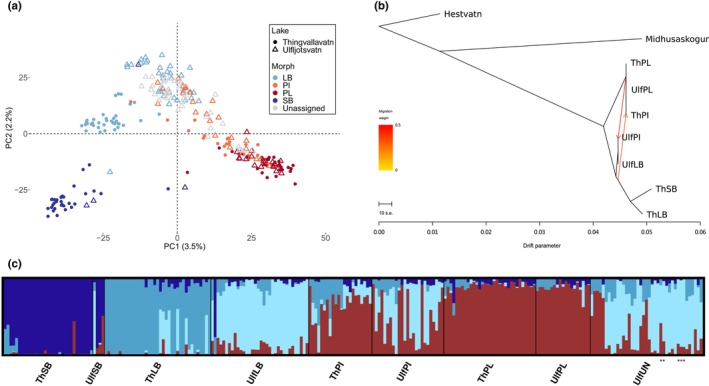
Population structure and admixture among sympatric charr morphs in the Thingvallavatn‐Ulfljotsvatn system based on 7840 genome‐wide SNPs from 243 individuals. (a) Principal component analysis showing the first and second PCs. (b) Genome‐wide admixture inferred with TreeMix with two migration edges (*m* = 2). (c) Admixture plots depicting the proportion of genetic ancestry for four (*K* = 4) genetic clusters. Stars indicate the ‘snail‐eating’ PL‐like charr.

A PCA revealed distinct genetic clustering of charr morphs within and between lakes. In both lakes, three of the four morphs (SB‐, LB‐, and PL‐charr) formed clear clusters (Figures 4a and S5). PC1 separated the Limnetic morphs from the benthic morphs, while PC2 distinguished SB‐ from LB‐charr. Many PI‐charr clustered with PL‐charr, though some were spread towards the LB‐charr cluster, with a few overlapping it, indicating potential mixing. Unassigned individuals from Ulfljotsvatn were genetically diverse, most overlapped with LB‐ and PI‐charr, and a few with PL‐charr in Ulfljotsvatn. When comparing clustering between lakes, PL‐charr from both lakes formed a single, distinct cluster. PI‐charr displayed similar genetic composition along the PL‐LB axis, but a much higher proportion of PI‐charr in Ulfljotsvatn clustered with LB‐charr than in Thingvallavatn. Interestingly, LB‐charr from Ulfljotsvatn were clearly separated from LB‐charr in Thingvallavatn. Evidence of misclassification and potential hybridisations was also detected: two SB‐charr and one LB‐charr caught in Ulfljotsvatn clustered with SB‐charr from Thingvallavatn, while one SB‐ and one PL‐charr from Thingvallavatn, along with one SB‐charr from Ulfljotsvatn, appear to be hybrids between PL‐ and SB‐charr. Additionally, the ‘snail‐eating’ PL‐like charr from Ulfljotsvatn clustered genetically with LB‐charr from the same lake.

In the admixture analysis, four, five and six clusters were identified as the most likely values for *K* using different evaluation methods (Figure S6a,b). At *K* = 4, four genetic groups emerged, corresponding to PL‐charr in both lakes, SB‐charr in Thingvallavatn (including two individuals caught in Ulfljotsvatn), and LB‐charr in each lake, which is consistent with the PCA results (Figure 4c). Hybridisation was supported in PI‐charr, as most individuals exhibited varying degrees of mixed ancestry between PL‐charr and the respective LB‐charr populations in each lake. Interestingly, most PI‐charr in Thingvallavatn showed high PL‐charr ancestry, whereas PI‐charr in Ulfljotsvatn had a greater proportion of LB‐charr ancestry. Individuals that appeared misclassified or as hybrids in the PCA also exhibited unusual ancestry patterns in the admixture analyses. Surprisingly, seven LB‐charr individuals in Thingvallavatn showed significant admixture (over 38%, with an average of 53%) with LB‐charr from Ulfljotsvatn, given that upstream migration from Ulfljotsvatn to Thingvallavatn is no longer feasible. Unassigned individuals from Ulfljotsvatn displayed a wide range of ancestries, corresponding to their position along the PL‐LB axis in the PCA. At *K* = 5 and 6, the additional ancestries did not correspond to any morph or locality, instead appearing in nearly all groups (Figure S6c).

Pairwise comparisons of *F*_ST_ between morphs (groups) within each lake ranged from 0.0172 (ThPL vs. ThPI) to 0.0543 (ThSB vs. ThLB) in Thingvallavatn and from 0.0100 (UlfLB vs. UlfUN) to 0.0532 (UlfSB vs. UlfPL) in Ulfljotsvatn (Table S6). Comparisons between the same morphs across lakes were consistent with the PCA and admixture analyses; LB‐charr showed the greatest differentiation (*F*_ST_ = 0.0269), while PL‐charr were the most similar (*F*_ST_ = 0.0120). The consistent clustering of four morph‐specific genetic groups, along with potential hybridisation, was also detected in a neighbour‐joining network (Figure S7). Notably, the seven ThLB‐charr individuals with a high proportion of UlfLB‐charr ancestry clustered closely with LB‐charr from Ulfljotsvatn.

### 3.7 Historical and Contemporary Gene Flow

To further evaluate gene flow and introgression among charr morphs at different levels, we used several approaches. TreeMix revealed historical gene flow exclusively within lakes, with no admixture detected between lakes, including reference populations (Figure 4b). In Thingvallavatn, gene flow was inferred from LB‐ to PI‐charr, while in Ulfljotsvatn admixture occurred between PL‐ and PI‐charr (Figure S8). In the combined analysis, the migration event in Ulfljotsvatn was consistently recovered, whereas in Thingvallavatn gene flow to PI‐charr was supported from the benthic morphs rather than from LB‐charr alone. Evidence for gene flow between LB‐ and PI‐charr within each lake was further corroborated by significant non‐zero D‐statistics (Table S7), and the f_4_‐branch metric highlighted the substantial proportion of introgression in each case (Figure S9). Notably, the across‐lake gene flow between LB‐charr inferred from the admixture analysis was also supported by the f_4_‐ratio (but not TreeMix).

To analyse contemporary gene flow, we used BA3‐SNPs to estimate migration rates between charr morphs within and between lakes (Table S8). Although migration rates between morphs within each lake were generally low and often close to their standard deviations, elevated values were detected between PI‐ and PL‐charr in Thingvallavatn and between LB‐ and PI‐charr in Ulfljotsvatn. Notably, inter‐morph migration appeared more pronounced in Ulfljotsvatn than in Thingvallavatn. Moreover, gene flow in these pairs was predominantly unidirectional: the mean migration rate from PI‐ to PL‐charr in Thingvallavatn was 0.037, compared to only 0.013 in the opposite direction. In Ulfljotsvatn, the rate from LB‐ to PI‐charr was relatively high at 0.119, while the reverse migration rate was 0.072. Inter‐lake migration rates between morphs were mostly low (0.007–0.01) and symmetrical. However, downstream migration from limnetic morphs in Thingvallavatn to morphs in Ulfljotsvatn was notably higher in some cases (e.g., ThPL to UlfPI reached 0.07).

### 3.8 Genetic Differentiation and Persistence of Sympatric Charr Morphs by Lake

**FIGURE 5:**
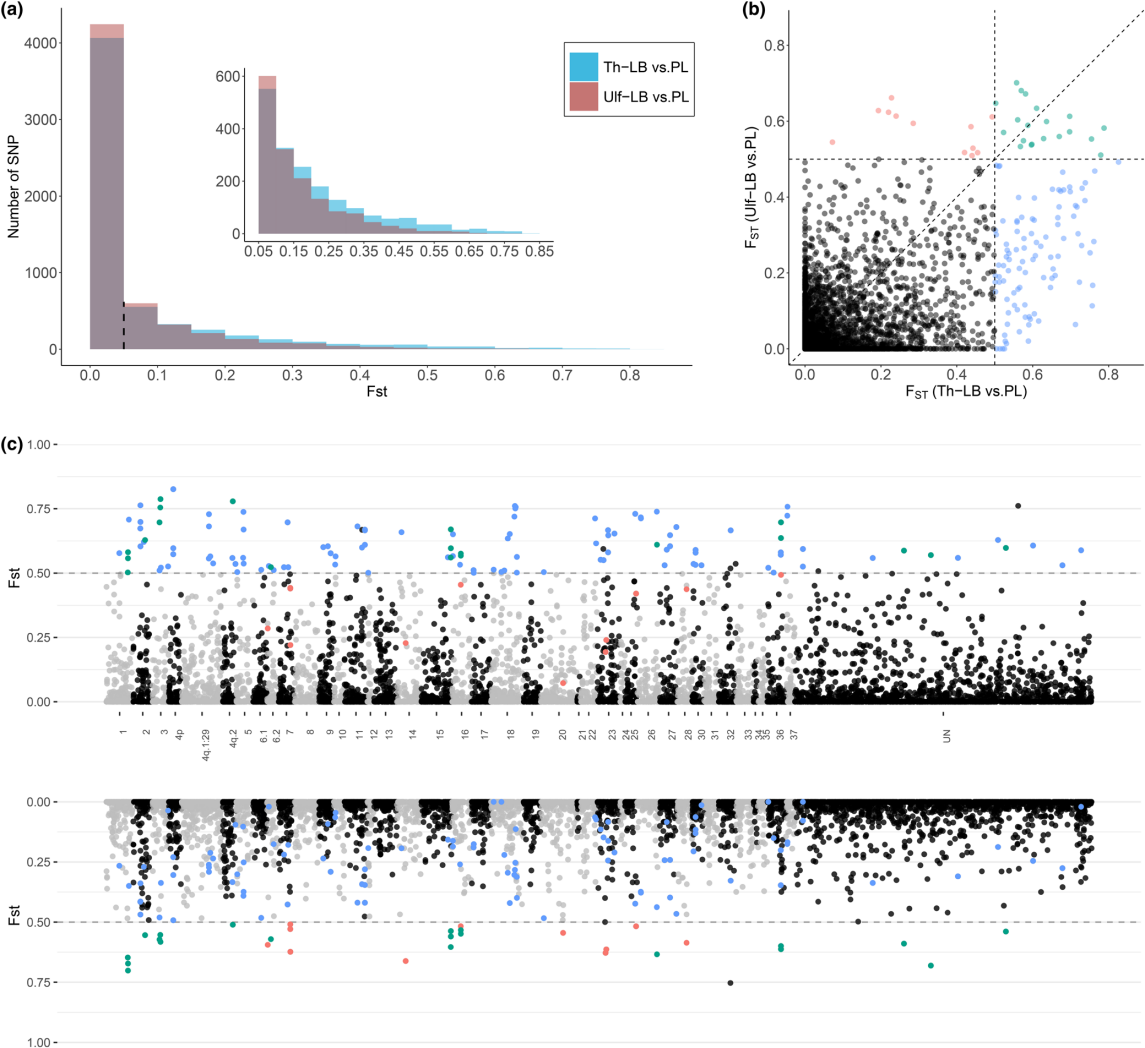
Genomic differentiation between LB‐ and PL‐charr in Thingvallavatn and Ulfljotsvatn. (a) Distribution of *F*_ST_ values between LB‐ and PL‐charr in Thingvallavatn (blue) and Ulfljotsvatn (red). (b) Genomic divergence between LB and PL‐charr across lakes based on *F*_ST_ of shared SNP markers, with highlighted Thingvallavatn‐specific *F*_ST_ outliers in blue, Ulfljotsvatn‐specific *F*_ST_ outliers in red, and outliers shared by both lakes in green. (c) SNP‐based *F*_ST_ between LB‐ and PL‐charr in Thingvallavatn (above) and Ulfljotsvatn (below) across the *Salvelinus* sp. genome. Outliers from shared SNPs detected in (b) were highlighted using the same colours.

We observed stronger genomic differentiation between LB‐ and PL‐charr in Thingvallavatn than in Ulfljotsvatn. Both the median *F*_ST_ and the number of markers with *F*_ST_ above 0.5 are higher in Thingvallavatn than in Ulfljotsvatn (Figure 5a). Out of 4611 SNPs common to both lakes, 98 had *F*_ST_ values above 0.5 only in Thingvallavatn, while only 12 SNPs had *F*_ST_ values above 0.5 exclusively in Ulfljotsvatn. Twenty SNPs with *F*_ST_ exceeding 0.5 were found in both lakes, distributed across 9 linkage groups (including unassigned contigs merged as ‘UN’) (Figure 5c). A total of 9 genes had at least one SNP within the transcribed region (Table 2).

**TABLE 2:** Summary of genes that were captured by 20 highly differentiated SNPs (*F*_ST_ > 0.5) between LB‐ and PL‐charr in Thingvallavatn and Ulfljotsvatn.

ID	Gene name	LG	Number of SNPs	GO function
111968723	Cadherin‐4‐like isoform X1	1	1	Enables cadherin binding/calcium ion binding
111951398	Ankyrin repeat and BTB/POZ domain‐containing protein BTBD11‐A‐like	3	1	Enables protein heterodimerization activity
111951675	Collagen alpha‐1 (XXV) chain isoform X1	3	1	NA
111951720	O‐phosphoseryl‐tRNA(Sec) selenium transferase	3	1	Enables O‐phosphoseryl‐tRNA(Sec) selenium transferase activity/tRNA binding
111963478	Tyrosine‐protein kinase receptor TYRO3	4q.2	1	Enables ATP binding/protein binding/transmembrane receptor protein tyrosine kinase activity
111974157	DENN domain‐containing protein 5A isoform X1	15	1	Enables guanyl‐nucleotide exchange factor activity/small GTPase binding
111975138	Switch‐associated protein 70‐like	15	2	NA
111975574	Cytosolic carboxypeptidase 6 isoform X1	16	2	Enables DNA binding/metallocarboxypeptidase activity/zinc ion binding
112072668	Elongin‐C‐like	UN	1	Enables protein‐macromolecule adaptor activity

## 4 Discussion

The repeated co‐occurrence of two or more Arctic charr morphs in watersheds throughout its Holarctic distribution range has spurred numerous studies on recent sympatric divergence (Brachmann et al. 2022; Jacobs et al. 2020; Salisbury et al. 2020). Key questions regarding how sympatric charr polymorphism persists with incomplete reproductive isolation and the role of spatial heterogeneity remain largely unexplored. In this study, we combined phenotypic and genetic data to assess the status of sympatric charr morphs in the Thingvallavatn‐Ulfljotsvatn system and examined whether these connected lakes harbour metapopulations of one or more morphs. Our findings confirmed the single origin of charr polymorphism in the system and the presence of all four charr morphs in Ulfljotsvatn. We evaluated the evidence for a metapopulation structure in each morph and found the situation to be morph specific. Differentiation between LB‐ and PL‐charr was lower in Ulfljotsvatn, potentially due to introgression from PI‐ to LB‐charr. These results suggest that the connection between the lakes has led to complex eco‐evolutionary dynamics of sympatric charr morphs across the system and played a special role in the maintenance of this recent sympatric divergence.

### 4.1 Charr Morphs in the Thingavallvatn‐Ulfljotsvatn System

Although charr morphs in Thingvallavatn have been well described, understanding the entire system requires thorough examination and careful comparisons of morphs across lakes. Phylogenetic reconstruction provided clear evidence that charr in the lake system form a monophyletic group, ruling out significant external gene flow. Considering the observed alignment of diet, morphology, and genetics, we can infer that the four ecologically distinct charr morphs in Thingvallavatn are also present in Ulfljotsvatn, and there is no support for independent evolution of this polymorphism in both lakes. Similar cases of highly diverse sympatric Arctic charr polymorphism across connected lakes are rare, with a notable exception in southwestern Greenland (Doenz et al. 2019). In two large lakes within the Eqaluit River drainage, five specialised ecomorphs coexist, four of which share comparable phenotypic and ecological traits with corresponding Thingvallavatn morphs. However, secondary contact and hybridisations between different glacial lineages of charr may have contributed to this diverse assemblage, and the impact of repeated colonisation cannot be ruled out (Brunner et al. 2001; Jacobsen et al. 2022). By contrast, charr morphs observed in the Thingvallavatn‐Ulfljotsvatn system represent a unique case of highly diverse sympatric polymorphism that likely evolved from a single colonisation followed by expansion into connected waterbodies and unoccupied niches.

Using genome‐wide SNPs, we confirmed that three of the four morphs, namely SB‐, LB‐ and PL‐charr, are genetically distinct. This finding is consistent with previous studies on charr morphs in Thingvallavatn using different types of markers (Brachmann et al. 2021; Guðbrandsson et al. 2019; Kapralova et al. 2011) and highlights the coupling of rapid adaptive divergence with emerging reproductive isolation. Our results also further emphasise the complex status of PI‐charr. Early studies indicated that juvenile PL‐ and PI‐charr were morphologically indistinguishable (Malmquist et al. 1985), leading to the hypothesis that PI‐charr are PL‐charr offspring that have gone through an ontogenetic niche shift after reaching a certain size and starting to prey on sticklebacks and juvenile charr (Jonsson et al. 1988; Snorrason et al. 1994). Consequently, PI‐charr would be expected to show minimal genetic differentiation from PL‐charr. However, both our current findings and a recent study found that many PI‐charr show genetic admixture with LB‐charr in addition to affinity with PL‐charr (Guðbrandsson et al. 2019). These results indicate that some PI‐charr may subsequently hybridise with LB‐charr. Alternatively, a non‐exclusive possibility is that some LB‐charr individuals may also transit into PI‐charr through ontogenetic shift. The observation that many PI‐charr from Ulfljotsvatn clustered genetically with LB‐charr in the same lake appears to support the latter scenario. Further work is necessary to explore the evolutionary and demographic history of PI‐charr.

### 4.2 Does the Thingavallvatn‐Ulfljotsvatn System Harbour Metapopulations of Charr Morphs?

Considering the geographic setting, and the differences in the relative sizes and quality of specific habitats between lakes, we expected to detect a definite source‐sink structure for one or more morphs in the connected lakes. We found that PL‐charr in Ulfljotsvatn were nearly indistinguishable from PL‐charr in Thingvallavatn, both morphologically and genetically, suggesting high downstream migration likely due to the large population size and considerable mobility of PL‐charr in Thingvallavatn (Sandlund et al. 1992; Snorrason, Jónasson, et al. 1992). Given the restricted pelagic zone in Ulfljotsvatn, the fitness of PL‐charr immigrants is likely too low to ensure survival. This assumption is further supported by the diet analysis as the most consumed prey items by Ulfljotsvatn PL‐charr are of benthic origin.

We only caught four SB‐charr in Ulfljotsvatn, two of which were sexually mature and genetically clustered with SB‐charr from Thingvallavatn. This low abundance of SB‐charr in Ulfljotsvatn can be attributed to both potentially limited downstream migration and low local survival. In Thingvallavatn, SB‐charr are restricted to the shallow littoral zone (Sandlund et al. 1992), and the estimated population size is much smaller than that of PL‐charr (Snorrason, Sandlund, and Jonsson 1992). Moreover, the fitness of Ulfljotsvatn SB‐charr is likely to be low, as substrates of uneroded lava or lava rubble, typically utilised by SB‐charr in Thingvallavatn, are limited in Ulfljotsvatn. Consequently, occasional immigrants of SB‐charr from Thingvallavatn are unlikely to sustain a viable population in Ulfljotsvatn.

Unlike PL‐ and SB‐charr, we observed weak but distinct genomic differentiation between LB‐charr in the two lakes, indicating low between‐lake genetic connectivity and the establishment of a local population in Ulfljotsvatn. The differences in mtDNA haplotype frequencies of LB‐charr between lakes are consistent with their nuclear genetic differentiation and suggest LB‐charr in each lake may be isolated from each other. Additionally, geometric morphometrics revealed shape and allometric differences between lakes, which could indicate local adaptation through genetic differentiation and/or plasticity.

Most curiously, we detected introgression from Ulfljotsvatn to Thingvallavatn in LB‐charr by Admixture, suggesting hidden interactions between lakes. Given the strong rapids in river Efra‐Sog and the construction of the dam in 1959, recent admixture due to upstream emigration to Thingvallavatn seems spurious. Importantly, the seven admixed individuals were caught outside the breeding season and far from the main spawning site of Thingvallavatn LB‐charr. Considering the large size of Thingvallavatn and the homing behaviour of spawning charr, we cannot rule out the presence of an unknown, genetically distinct LB‐charr population within Thingvallavatn. Although the cold spring area at Olafsdrattur is the only well studied spawning site of LB‐charr in Thingvallavatn (Horta‐Lacueva et al. 2022; Sigurjónsdóttir and Gunnarsson 1989), the littoral zone along the northern and eastern shores no doubt harbours several cold spring areas, which could serve as additional breeding sites. In the 1970s, divers observed spawning LB‐charr at a depth of 4 m near cold water springs about 2 km north of the outflow (Sandlund et al. 1992). Assuming there is a distinct LB‐charr population spawning in this area, the seven admixed individuals could represent hybrids between this putative population and the Olafsdrattur population. Moreover, LB‐charr in Ulfljotsvatn may have been established by founders from this population. Further studies are needed to test this hypothesis.

We were unable to disentangle the relationship of PI‐charr between lakes due to the absence of a distinct genetic cluster for this morph. Overall, our findings suggest that the connected lakes function as a source‐sink system (Kawecki 2004) for PL‐ and SB‐charr, with Ulfljotsvatn acting as an ecological ‘cul de sac’ for these morphs. However, the between‐lake dynamics of these two morphs may vary considerably. Fishing surveys conducted between 1993 and 2000 reported consistently low relative abundance of SB‐charr (0.8%–1.8%) in Ulfljotsvatn, but with occasional spikes (43% in 1994 and 23% in 1996) (B. Jónsson et al. 2021). These fluctuations in population size suggest that temporal variations in passive dispersal of SB‐charr from Thingvallavatn may drive a pattern of regular extinction‐recolonisation in Ulfljotsvatn. While it remains unclear whether PL‐charr can enjoy some reproductive success in Ulfljotsvatn, the high immigration rate from Thingvallavatn likely substantially mitigates the risk of local extinction. Lastly, for LB‐charr, the system may more resemble a mainland‐island system with minimal contemporary gene flow between the lakes (Macarthur and Wilson 1967).

### 4.3 Coexistence of Sympatric Charr Morphs in the Lake System

From an eco‐evolutionary perspective, both resource competition and hybridisation among sympatric morphs are important criteria for determining the state of local coexistence (Weber and Strauss 2016). Apparently, PL‐charr are forced to compete with LB‐charr in Ulfljotsvatn, a situation not seen in Thingvallavatn (Malmquist et al. 1992). Moreover, we uncovered varying patterns of hybridisation among sympatric charr morphs across lakes. We identified three likely hybrids between SB‐ and PL‐charr in both lakes. The small number of sexually mature hybrids suggests that hybridisation between these morphs may occur at a low rate, or that hybrids have poor survival. Occasional hybridisation between SB‐ and LB‐charr may also happen in Thingvallavatn, as admixture analysis revealed LB‐charr ancestry in two SB‐charr individuals.

We found clear evidence of hybridisation between LB‐ and PI‐charr in both lakes. This was significantly stronger in Ulfljotsvatn, potentially due to its small size and reduced habitat heterogeneity. Similarly, introgression among several Galápagos ground finch species has been shown to be stronger on small, isolated peripheral islands than on centrally located, larger or more interconnected ones (Farrington et al. 2014). Given the distinct mismatch in peak spawning periods of these two morphs (Sandlund et al. 1992; Skúlason et al. 1989), the hybridisation is most likely explained by opportunistic breeding between LB‐charr males and PI‐charr females. Early surveys revealed that approximately 10% of LB‐charr males remained in maturity stages 5–6 in mid‐September, with numbers gradually decreasing until November (Skúlason et al. 1989). These LB‐charr males may seek to mate with PI‐charr females, likely succeeding due to similarities in size. Further field observations and mating experiments are needed to confirm this hypothesis.

The higher level of hybridisation between these two large morphs in Ulfljotsvatn may be due to reduced segregation in spawning sites or increased overlap of spawning periods. Considering the much smaller size of Ulfljotsvatn, the former seems more plausible. However, these scenarios are not mutually exclusive and require further investigation. While some of the unassigned individuals found in Ulfljotsvatn may represent recent hybrids, others that are genetically distinct may have shifted phenotypes due to plasticity. Additionally, we cannot rule out the possibility that the substantial difference in mtDNA haplotype frequencies observed between LB‐charr in the two lakes also reflects hybridisation. Moreover, the erosion of genetic differentiation between LB‐ and PL‐charr in Ulfljotsvatn is likely a side effect of introgression from PI‐ to LB‐charr. Direct hybridisation between PL‐ and LB‐charr is highly unlikely due to their different body sizes and limited temporal overlap in spawning (Sandlund et al. 1992). The shared SNPs with high *F*_ST_ across lakes suggest that some markers have persisted despite gene flow and may potentially act as barrier loci (Ravinet et al. 2017).

### 4.4 Implications

The introduction of the metapopulation concept pioneered the exploration of ecological patterns and processes underlying spatially structured populations (Hanski and Simberloff 1997). By integrating population genomics, we can now examine the evolutionary impacts of interpopulation dynamics on intraspecific differentiation, local adaptation, and speciation (Lamy et al. 2012; Manier and Arnold 2005; Thia et al. 2021). Both theoretical models and empirical studies demonstrate that metapopulations can evolve differently from large, single populations (Angst et al. 2022; Freitas et al. 2022). However, formulating general predictions remains challenging due to the high variability in metapopulation structure, migration rates between local populations, and the complexity of their interactions.

Our study examined a unique system involving two connected lakes that host four sympatric Arctic charr morphs in early stages of divergence. We found that compared to the relatively stable coexistence of sympatric morphs in Thingvallavatn, the maintenance of polymorphism in Ulfljotsvatn critically relies on downstream migration, mainly driven by the geographic configuration, which counteracts weakened reproductive isolation and the sorting effects of reduced habitat heterogeneity. Recent studies have examined the roles of reproductive interactions (Gómez‐Llano et al. 2021; Irwin and Schluter 2022) and metapopulation structure (Luo et al. 2022; Proulx et al. 2025) in species coexistence. Our results support the integration of these aspects into the coexistence theory and further highlight the importance of exploring their combined influence on coexistence. Another significant insight is how the interplay between habitat heterogeneity and morph‐specific migrations may have shaped the ecological relationships, especially the degree of hybridisation, among sympatric morphs in different lakes. Our data imply that the higher hybridisation of LB‐ and PI‐charr in Ulfljotsvatn could influence ongoing divergence and potentially alter the dynamics of coexistence among charr morphs. The role of hybridisation in coexistence and speciation is highly variable (Abbott et al. 2013; Irwin and Schluter 2022; Moran et al. 2021). Whether the observed hybridisation will slow ongoing divergence, lead to a hybrid swarm, or foster the emergence of a new lineage remains uncertain but warrants further attention.

The long‐term effects of metapopulation structure on sympatric divergence are largely unknown. While metapopulation structures have been observed in several well‐known systems of sympatric polymorphism at present (e.g., Hudson et al. 2011; Salisbury et al. 2023), it is challenging to confirm their presence in the past (but see Martin and Feinstein 2014). Compared to a single population, a metapopulation may persist longer by comprising several semi‐connected local populations, thus providing greater opportunities for genomic reorganisation through gene flow among local populations in varying spatiotemporal conditions (Levin 1995). In some instances, historical metapopulation dynamics may have facilitated sympatric speciation. For example, two small Nicaraguan crater lakes harbour six and four sympatric Midas cichlids, respectively. Both lakes, along with other surrounding crater lakes, have been repeatedly colonised by two species of Midas cichlids from two old and great lakes. This suggests that the great lakes and crater lakes formed interim source‐sink metapopulation systems. Demographic reconstructions strongly support an admixture event from each great lake into its corresponding crater lake prior to species divergence (Kautt, Machado‐Schiaffino, and Meyer 2016). Overall, our findings underscore the crucial utility of early‐diverging morphs in eco‐evolutionary research and highlight the significance of studying metapopulation structures to deepen our understanding of biodiversity, particularly in contexts of increasing complexity and ecological realism.

## Author Contributions

S.S.S., H.X., Z.O.J. and A.P. conceived of the study. All authors were responsible for sampling. S.S.S. and H.X. performed morph classification. Z.O.J. developed the genotype and primary analyses pipeline. A.P. designed the KASP markers. H.X. conducted the laboratory work, analysed data and wrote the original draft. All authors contributed to revisions of the manuscript.

## Funding

This research was funded by the Icelandic Research Fund (Grant number 152536‐052 to S.S.S. and Grant number 195908‐051 to H.X.), Landsvirkjun, and a Teaching Assistant Scholarship from the University of Iceland to H.X.

## Ethics Statement

Fish were sampled from the wild with permissions from the Directory of Fisheries, the farmers in Mjoanes, the Thingvellir National Park Commission, and landowners at Kaldarhofdi. Ethics committee approval is not needed for regular or scientific fishing in Iceland (The Icelandic law on Animal protection, Law 15/1994, last updated with Law 55/2013).

## Conflicts of Interest

The authors declare no conflicts of interest.

## Supporting information


**Data S1:** mec70225‐sup‐0001‐Supinfo.docx.

## Data Availability

Raw sequence reads have been deposited in the NCBI SRA under BioProject PRJNA1335803. Data underlying the genomic and geometric morphometric analyses, including input files and relevant metadata, have been deposited in Dryad and are available at: https://doi.org/10.5061/dryad.9cnp5hqvv.
