# Supplementary material for: Gene Flow and Habitat Heterogeneity Shape Coexistence Dynamics of Arctic Charr Morphs in Connected Lakes
Source: Mol Ecol. 2026 Jan 16;35(2):e70225. doi: 10.1111/mec.70225 (PMC12809625; doi:10.1111/mec.70225)
Supplement: Supplementary file 1 — Data S1: mec70225‐sup‐0001‐Supinfo.docx. [file MEC-35-e70225-s001.docx]

**Supplemental Information for:**

**Gene flow and habitat heterogeneity shape coexistence dynamic of Arctic charr morphs in connected lakes**

**Han Xiao^1^ |** **Arnar Pálsson ^1^ | Zophonías O. Jónsson ^1^ | Sigurður S. Snorrason ^1^**

**Table of Contents:**

| **Table S1.** Information for KASP assays | | Page 1 |
| --- | --- | --- |
| **Table S2.** The occurrence of diet items in stomachs of Arctic charr morphs in Ulfljotsvatn | | Page 2 |
| **Table S3.** Summary for analyses using linear models to investigate associations between variables and head shape. | | Page 3 |
| **Table S4.** Summary for analyses using linear models to test for allometry. | | Page 4 |
| **Table S5.** Number of SNPs remaining after sequential filtration steps. | | Page 5 |
| **Table S6.** Pairwise *F*_ST_ between morphs within and between lakes based on genome-wide SNPs. | | Page 6 |
| **Table S7.** Detailed results of Patterson's D statistic and f_4_- ratio test for selected trios | | Page 7 |
| **Table S8.** Recent migration rates among sympatric morphs. | | Page 8 |
| **Figure S1.** The landmarks and semi-landmarks used for geometric morphometric analysis. | | Page 9 |
| **Figure S2.** Allometric relationship between head shape of sympatric morphs and centroid size | | Page 9 |
| **Figure S3.** Genetic connectivity and phylogeography of Arctic charr in the lake system and reference populations | | Page 10 |
| **Figure S4.** Stacked bar chart showing the number of individuals carrying unique haplotypes. | Page 10 |  |
| **Figure S5.** Principal component analysis of charr morphs showing PC3 and PC4. | Page 11 |  |
| **Figure S6.** Additional information for admixture analyses. | | Page 12 |
| **Figure S7.** Neighbor-Net network of charr morphs in the connected lakes. | | Page 13 |
| **Figure S8.** Additional information for tree graphs with migration edges inferred by Treemix. | | Page 14 |
| **Figure S9.** Results of Fbranch for the charr morphs and reference populations | Page 15 |  |

**Supplementary Table 1.** Information for KASP assays used in this study.

| Assays ID | Allele (FAM/HEX) | Sequence (5'-3') |
| --- | --- | --- |
| m1829 | G/A | TAAGACCCAAAACGTCAGGTCGAGGTGTAGCGCATGGGGTGGGAAGAAATGGGCTACATTCTCTAAATTAGAGCACTACGAACCACGTTGTGAAACCAAC[target SNP allele] TCCGAAGGTGGATTTAGCAGTAAACAGAAAACAGAGAGTTCTCTTGAAACTGGCTCTGAGGCGCGCACACACCGCCCGTCACTCTCCCCAAGTTTAATTT |
| m3211 | T/C | TCTTTTAAATGAAGACCTGTATGAATGGCATCACGAGGGCTTAGCTGTCTCCTCTCCCAAGTCAATGAAATTGATCTGCCCGTGCAGAAGCGGGCATAAG[target SNP allele]  ACATAAGACGAGAAGACCCTATGGAGCTTTAGACACCAGGCAGATCACGTCAAGTAAACTTAAATTAACAAGTAAAAACGCAGTGACCCCTAGCCCATAT |

**Supplementary Table 2.** The occurrence of diet items in stomachs of Arctic charr morphs in Ulfljotsvatn by a) specific numbers (N) and b) frequency (%).
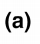


| Morph | Empty stomach | From benthic habitat | | | | | | | |  |  |  |  | Total |
| --- | --- | --- | --- | --- | --- | --- | --- | --- | --- | --- | --- | --- | --- | --- |
|  |  | Stones | Snail^1^ | Benthic crustacea | Trichoptera^2^ | Chironomid larvae | Other insects^3^ | Algae | Eggs^4^ | Zoo-  plankton | Terrestrial invertebrates | Insect remains^5^ | Digested remains^5^ |  |
| LB | 7 | 5 | 63 | 12 | 3 | 10 | 0 | 0 | 3 | 1 | 0 | 16 | 4 | 84 |
| PI | 19 | 1 | 1 | 2 | 0 | 0 | 0 | 2 | 4 | 1 | 0 | 1 | 1 | 28 |
| PL | 16 | 0 | 5 | 8 | 2 | 7 | 0 | 3 | 1 | 5 | 2 | 7 | 1 | 42 |
| SB | 1 | 0 | 1 | 0 | 0 | 0 | 0 | 0 | 2 | 0 | 0 | 0 | 0 | 4 |
| UN | 12 | 3 | 39 | 14 | 10 | 11 | 1 | 4 | 6 | 3 | 2 | 6 | 3 | 74 |


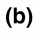


| Morph | Empty stomach | From benthic habitat | | | | | | | |  |  |  |  |
| --- | --- | --- | --- | --- | --- | --- | --- | --- | --- | --- | --- | --- | --- |
|  |  | Stones | Snail^1^ | Benthic crustacea | Trichoptera^2^ | Chironomid larvae | Other insects^3^ | Algae | Eggs^4^ | Zoo-  plankton | Terrestrial invertebrates | Insect remains | Digested remains |
| LB | 8.3 | 6.0 | 75.0 | 14.3 | 3.6 | 11.9 | 0 | 0 | 3.6 | 1.2 | 0 | 19.0 | 4.8 |
| PI | 67.9 | 3.6 | 3.6 | 7.1 | 0 | 0 | 0 | 7.1 | 14.3 | 3.6 | 0 | 3.6 | 3.6 |
| PL | 38.1 | 0 | 11.9 | 19.0 | 4.8 | 16.7 | 0 | 7.1 | 2.4 | 11.9 | 4.8 | 16.7 | 2.4 |
| SB | 25.0 | 0 | 25.0 | 0 | 0 | 0 | 0 | 0 | 50.0 | 0 | 0 | 0 | 0 |
| UN | 16.2 | 4.1 | 52.7 | 18.9 | 13.5 | 14.9 | 1.4 | 5.4 | 8.1 | 4.1 | 2.7 | 8.1 | 4.1 |

1. *Radix pergra*; 2. Larvae and pupae; 3. Only larvae; 4. From salmonid. 5. Unidentified

**Supplementary Table 3.** Summary of four different linear models used to test for associations between variables (morph, lake, and unique morph) and head shape (Procrustes coordinates hereafter) of charr morphs in Thingvallavatn and Ulfljotsvatn, including all possible interactions. Morph contains the four morphs regardless of lake of origin. Unique morph refers to each of the four morphs in each lake separately. Threshold of significance was determined using a residual randomization permutation procedure with 10 000 iterations.

| Model parameter | Df | SS | MS | R^2^ | F | Z | Pr(>F) |
| --- | --- | --- | --- | --- | --- | --- | --- |
| Shape~morph |  |  |  |  |  |  |  |
| morph | 3 | 0.46998 | 0.15666 | 0.29377 | 33.555 | 9.5722 | 0.001 |
| Residuals | 242 | 1.12983 | 0.004669 | 0.70623 |  |  |  |
| Total | 245 | 1.59981 |  |  |  |  |  |
| Shape ~ lake |  |  |  |  |  |  |  |
| lake | 1 | 0.03321 | 0.033208 | 0.02076 | 5.1721 | 3.326 | 0.001 |
| Residuals | 244 | 1.5666 | 0.006421 | 0.97924 |  |  |  |
| Total | 245 | 1.59981 |  |  |  |  |  |
| Shape~ morph×lake |  |  |  |  |  |  |  |
| lake | 1 | 0.03321 | 0.033208 | 0.02076 | 7.834 | 4.0938 | 0.001 |
| morph | 3 | 0.4798 | 0.159933 | 0.29991 | 37.7295 | 8.9507 | 0.001 |
| lake:morph | 3 | 0.07794 | 0.02598 | 0.04872 | 6.1289 | 5.9645 | 0.001 |
| Residuals | 238 | 1.00887 | 0.004239 | 0.63062 |  |  |  |
| Total | 245 | 1.59981 |  |  |  |  |  |
| Shape~unique morph |  |  |  |  |  |  |  |
| unique_morph | 7 | 0.59095 | 0.084421 | 0.36938 | 19.916 | 12.45 | 0.001 |
| Residuals | 238 | 1.00887 | 0.004239 | 0.63062 |  |  |  |
| Total | 245 | 1.59981 |  |  |  |  |  |

**Supplementary Table 4.** Summary of four linear models used to test for allometry (the covariation between head shape and log-transformed centroid size) across morph, morph and lake, and unique morph in Thingvallavatn and Ulfljotsvatn, with all possible interactions included. Threshold of significance was determined using a residual randomization permutation procedure with 10 000 iterations.

|  | Df | SS | MS | R^2^ | F | Z | Pr(>F) |
| --- | --- | --- | --- | --- | --- | --- | --- |
| Shape~log(Csize) |  |  |  |  |  |  |  |
| log(Csize) | 1 | 0.36267 | 0.36267 | 0.22669 | 71.528 | 8.054 | 0.001 |
| Residuals | 244 | 1.23714 | 0.00507 | 0.77331 |  |  |  |
| Total | 245 | 1.59981 |  |  |  |  |  |
| Shape~log(Csize)*morph |  |  |  |  |  |  |  |
| log(Csize) | 1 | 0.36267 | 0.36267 | 0.22669 | 86.6474 | 8.3812 | 0.001 |
| morph | 3 | 0.21308 | 0.07103 | 0.13319 | 16.9692 | 8.7121 | 0.001 |
| log(Csize):morph | 3 | 0.0279 | 0.0093 | 0.01744 | 2.2222 | 2.7676 | 0.003 |
| Residuals | 238 | 0.99616 | 0.00419 | 0.62268 |  |  |  |
| Total | 245 | 1.59981 |  |  |  |  |  |
| Shape~log(Csize)*lake*morph |  |  |  |  |  |  |  |
| log(Csize) | 1 | 0.36267 | 0.36267 | 0.22669 | 93.0944 | 8.5114 | 0.001 |
| lake | 1 | 0.03311 | 0.03311 | 0.0207 | 8.4996 | 4.9446 | 0.001 |
| morph | 3 | 0.19724 | 0.06575 | 0.12329 | 16.8771 | 8.7962 | 0.001 |
| log(Csize):lake | 1 | 0.02913 | 0.02913 | 0.01821 | 7.4772 | 4.9504 | 0.001 |
| log(Csize):morph | 3 | 0.02775 | 0.00925 | 0.01735 | 2.3743 | 3.149 | 0.002 |
| lake:morph | 3 | 0.03624 | 0.01208 | 0.02265 | 3.1007 | 3.9945 | 0.001 |
| log(Csize):lake:morph | 3 | 0.01766 | 0.00589 | 0.01104 | 1.5111 | 1.6105 | 0.055 |
| Residuals | 230 | 0.89601 | 0.0039 | 0.56007 |  |  |  |
| Total | 245 | 1.59981 |  |  |  |  |  |
| Shape~log(Csize)*unique_morph |  |  |  |  |  |  |  |
| log(Csize) | 1 | 0.36267 | 0.36267 | 0.22669 | 93.0944 | 8.5114 | 0.001 |
| unique_morph | 7 | 0.28444 | 0.04063 | 0.17779 | 10.4305 | 10.2778 | 0.001 |
| log(Csize):unique_morph | 7 | 0.0567 | 0.0081 | 0.03544 | 2.0791 | 3.8514 | 0.001 |
| Residuals | 230 | 0.89601 | 0.0039 | 0.56007 |  |  |  |
| Total | 245 | 1.59981 |  |  |  |  |  |

**Supplementary Table 5.** Number of SNPs remaining after sequential filtration steps for a) “global” dataset and b) “regional” dataset.

| Filters in populations | SNP count |
| --- | --- |
| Genotyped in more than : |  |
| 7/11 populations (-p 7) and |  |
| 2/3 of individuals in a population (-r 0.66) | 102146 |
|  |  |
| Maximum observed heterozygosity to be 0.60 (--max-obs-het 0.60) | 100985 |
|  |  |
| Minimum minor allele count to be 3 (--min-mac 3) | 23659 |
|  |  |
| First SNP of each locus (--write-single-snp) | 11160 |


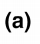


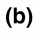


| Filters in populations | SNP count |
| --- | --- |
| Genotyped in more than : |  |
| 6/9 populations (-p 6) and |  |
| 2/3 of individuals in a population (-r 0.66) | 87761 |
|  |  |
| Maximum observed heterozygosity to be 0.60 (--max-obs-het 0.60) | 86486 |
|  |  |
| Minimum minor allele count to be 3 (--min-mac 3) | 16005 |
|  |  |
| First SNP of each locus (--write-single-snp) | 7840 |

**Supplementary Table 6.** Weir and Cockerham’s pairwise *F*_ST_ between morphs within and between lakes based on genome-wide SNPs. Comparisons between same morphs across lakes are highlighted in light grey.

| Unique morph | ThSB | UlfSB | ThLB | UlfLB | ThPI | UlfPI | ThPL | UlfPL | UlfUN |
| --- | --- | --- | --- | --- | --- | --- | --- | --- | --- |
| ThSB | --- | 0.0253 | 0.0330 | 0.0380 | 0.0485 | 0.0452 | 0.0543 | 0.0578 | 0.0381 |
| UlfSB |  | --- | 0.0315 | 0.0247 | 0.0382 | 0.0325 | 0.0339 | 0.0532 | 0.0222 |
| ThLB |  |  | --- | 0.0269 | 0.0390 | 0.0371 | 0.0493 | 0.0517 | 0.0281 |
| UlfLB |  |  |  | --- | 0.0248 | 0.0169 | 0.0336 | 0.0345 | 0.0100 |
| ThPI |  |  |  |  | --- | 0.0183 | 0.0172 | 0.0204 | 0.0184 |
| UlfPI |  |  |  |  |  | --- | 0.0205 | 0.0225 | 0.0112 |
| ThPL |  |  |  |  |  |  | --- | 0.0120 | 0.0258 |
| UlfPL |  |  |  |  |  |  |  | --- | 0.0254 |

**Supplementary Table 7.** Detailed results of Patterson's D statistic (ABBA- BABA test) and f_4_- ratio test conducted using Dsuite. Only tests with Z-scores greater than 3 and p-values less than 0.001 are included, indicating statistically significant signals of introgression.

| **P1** | **P2** | **P3** | **Dstatistic** | **Z-score** | **p-value** | **f4-ratio** | **BBAA** | **ABBA** | **BABA** |
| --- | --- | --- | --- | --- | --- | --- | --- | --- | --- |
| UlfPL | ThLB | Midh | 0.0467 | 3.0033 | 0.0013 | 0.0333 | 521.654 | 170.025 | 154.855 |
| ThPL | UlfLB | Midh | 0.0402 | 3.2188 | 0.0006 | 0.0285 | 543.863 | 171.953 | 158.668 |
| ThPL | UlfPI | Midh | 0.0407 | 4.4712 | 0.0000 | 0.0278 | 545.909 | 165.413 | 152.469 |
| ThPL | UlfLB | Midh | 0.0402 | 3.2188 | 0.0006 | 0.0285 | 543.863 | 171.953 | 158.668 |
| ThPL | UlfPI | Midh | 0.0407 | 4.4712 | 0.0000 | 0.0278 | 545.909 | 165.413 | 152.469 |
| UlfPL | ThPI | ThLB | 0.0524 | 6.9674 | 0.0000 | 0.1981 | 221.114 | 214.112 | 192.773 |
| ThPL | UlfPI | ThLB | 0.0426 | 6.6837 | 0.0000 | 0.1704 | 238.63 | 241.336 | 221.598 |
| ThSB | ThLB | UlfLB | 0.0497 | 4.6067 | 0.0000 | 0.8894 | 262.318 | 240.276 | 217.511 |
| ThSB | ThLB | UlfPI | 0.0375 | 3.1742 | 0.0008 | 0.9597 | 276.584 | 223.623 | 207.462 |
| UlfPL | UlfPI | ThLB | 0.0461 | 5.3818 | 0.0000 | 0.1801 | 229.98 | 231.107 | 210.729 |
| ThPL | ThPI | ThSB | 0.0319 | 4.5743 | 0.0000 | 0.1107 | 249.694 | 225.827 | 211.848 |
| ThPL | ThPI | UlfLB | 0.0378 | 5.6673 | 0.0000 | 0.3153 | 235.277 | 221.717 | 205.568 |
| UlfPI | ThPL | ThPI | 0.0252 | 3.0646 | 0.0011 | 0.7970 | 208.189 | 220.846 | 209.986 |
| UlfPL | ThPI | ThSB | 0.0329 | 3.8256 | 0.0001 | 0.1130 | 244.019 | 220.659 | 206.589 |
| UlfPL | ThPI | UlfLB | 0.0361 | 5.0506 | 0.0000 | 0.2986 | 229.291 | 215.474 | 200.475 |
| ThPL | UlfPI | ThSB | 0.0272 | 3.7934 | 0.0001 | 0.0966 | 265.649 | 252.999 | 239.59 |
| ThPL | UlfPI | UlfLB | 0.0515 | 6.4742 | 0.0000 | 0.4419 | 245.228 | 253.365 | 228.564 |
| ThSB | UlfLB | UlfPI | 0.0406 | 3.1808 | 0.0007 | 1.2622 | 264.18 | 256.406 | 236.4 |
| UlfPL | UlfPI | ThSB | 0.0306 | 3.6808 | 0.0001 | 0.1074 | 257.352 | 243.263 | 228.83 |
| UlfPL | UlfPI | UlfLB | 0.0521 | 5.6682 | 0.0000 | 0.4466 | 236.383 | 242.287 | 218.274 |

Note: Columns P1, P2 and P3 indicate the charr morphs/populations assigned to each position of the (((P1,P2),P3),OG) topology for each test, with Hestvatn as the outgroup (OG). The order of P1 and P2 is automatically arranged by Dsuite to ensure that nABBA >= nBABA, thereby producing positive D statistic values. Columns BBAA, ABBA and BABA represent the counts of derived alleles shared by (P1, P2), (P2, P3), and (P1, P3) for each of these site patterns under the given topology. The Z-score was calculated as Z=D/std_err(D) using a standard block-jackknife approach and the associated p-values were listed. The f_4_-ratio estimates the proportion of the genome involved in introgression.

**Supplementary Table 8.** Recent migration rates (the proportions of migrants each generation ± standard deviation) among abundant sympatric morphs (UlfSB excluded). The morphs giving migrants are listed in the columns, while the morphs receiving migrants are listed in the rows. (a) Migration rate among all charr morphs in Thingvallavatn. (b) Migration rate among abundant charr morphs in Ulfljotsvatn. (c). Migration rate among abundant charr morphs in Thingvallavatn and Ulfljotsvatn.


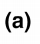


|  | ThLB | ThPI | ThPL | ThSB |
| --- | --- | --- | --- | --- |
| ThLB | 0.9756(0.0134) | 0.0081(0.0079) | 0.0082(0.0080) | 0.0082(0.0079) |
| ThPI | 0.0128(0.0124) | 0.9616(0.0205) | 0.0128(0.0123) | 0.0128(0.0124) |
| ThPL | 0.0093(0.0090) | 0.0369(0.0172) | 0.9353(0.0217) | 0.0185(0.0126) |
| ThSB | 0.0095(0.0092) | 0.0095(0.0092) | 0.0191(0.0130) | 0.9619(0.0177) |


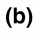


|  | UlfLB | UlfPI | UlfPL |
| --- | --- | --- | --- |
| UlfLB | 0.9189(0.0232) | 0.0721(0.0222) | 0.0090(0.0088) |
| UlfPI | 0.1190(0.0297) | 0.8691(0.0302) | 0.0119(0.0115) |
| UlfPL | 0.0151(0.0144) | 0.0152(0.0145) | 0.9697(0.0199) |


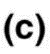


|  | ThLB | ThPI | ThPL | ThSB | UlfLB | UlfPI | UlfPL |
| --- | --- | --- | --- | --- | --- | --- | --- |
| ThLB | 0.9547(0.0169) | 0.0075(0.0074) | 0.0076(0.0074) | 0.0075(0.0074) | 0.0075(0.0074) | 0.0076(0.0074) | 0.0075(0.0073) |
| ThPI | 0.0114(0.0111) | 0.9308(0.0247) | 0.0116(0.0111) | 0.0115(0.0112) | 0.0115(0.0111) | 0.0115(0.0111) | 0.0116(0.0112) |
| ThPL | 0.0085(0.0083) | 0.0171(0.0116) | 0.9316(0.0212) | 0.0086(0.0084) | 0.0085(0.0083) | 0.0172(0.0117) | 0.0084(0.0083) |
| ThSB | 0.0088(0.0085) | 0.0088(0.0085) | 0.0176(0.0120) | 0.9386(0.0207) | 0.0087(0.0085) | 0.0088(0.0086) | 0.0089(0.0086) |
| UlfLB | 0.0081(0.0079) | 0.0406(0.0168) | 0.0082(0.0080) | 0.0082(0.0080) | 0.8943(0.0239) | 0.0325(0.0152) | 0.0082(0.0080) |
| UlfPI | 0.0104(0.0101) | 0.0104(0.0101) | 0.0729(0.0239) | 0.0104(0.0101) | 0.1042(0.0267) | 0.7812(0.0275) | 0.0104(0.0101) |
| UlfPL | 0.0128(0.0123) | 0.0127(0.0123) | 0.0383(0.0205) | 0.0128(0.0123) | 0.0128(0.0123) | 0.0128(0.0123) | 0.8976(0.0297) |


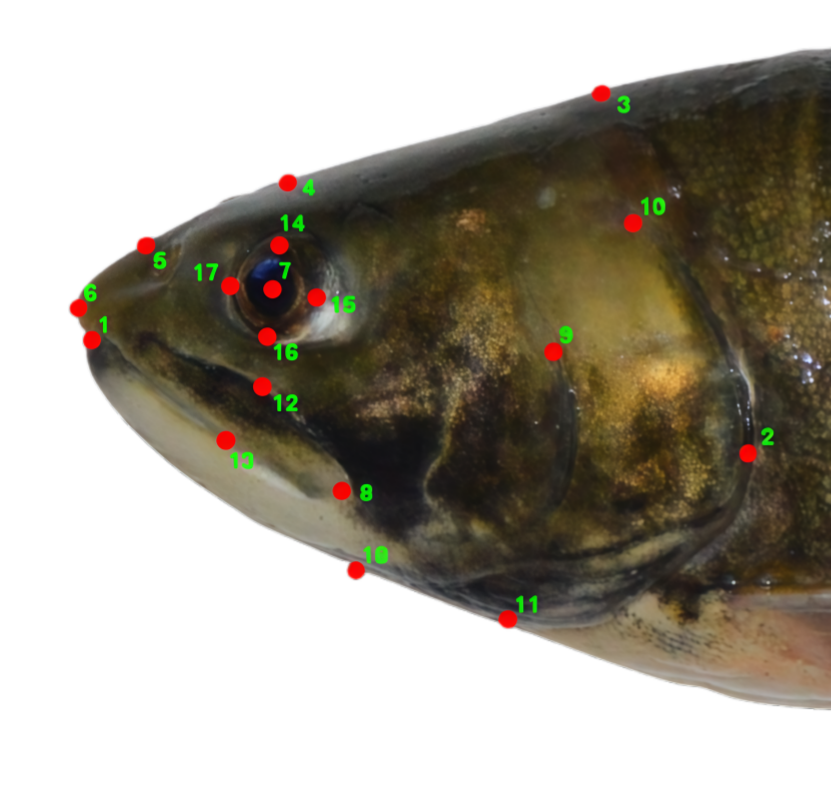


**Supplemental Figure S1.** The landmarks and semi-landmarks (4, 5, 11, 12, 13, 18) used for geometric morphometric analysis of head shape.


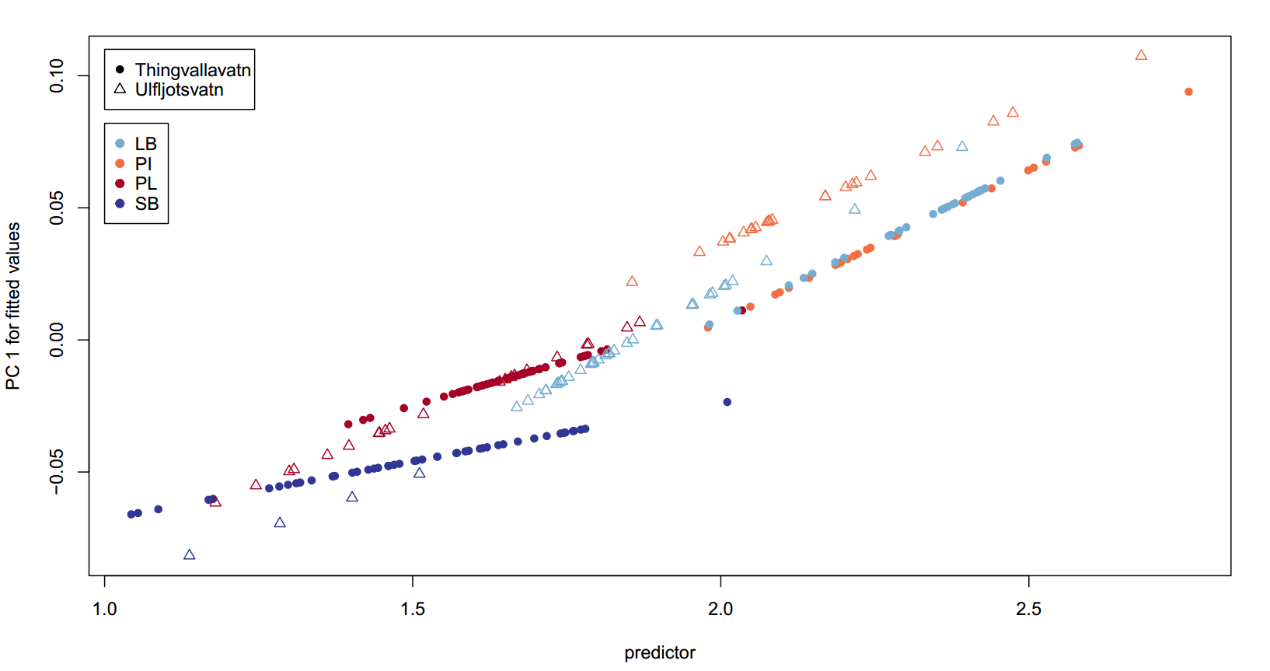


**Supplemental Figure S2.** Allometric relationship between head shape and centroid size of sympatric morphs in Thingvallavatn and Ulfljotsvatn, visualized as the log transformed CS along X-axis (predictor) strongly associated with the first principal component of the predicted values along Y-axis from a multivariate regression. The lakes are represented by symbols and morph assignment by colours (see legend).


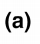

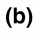

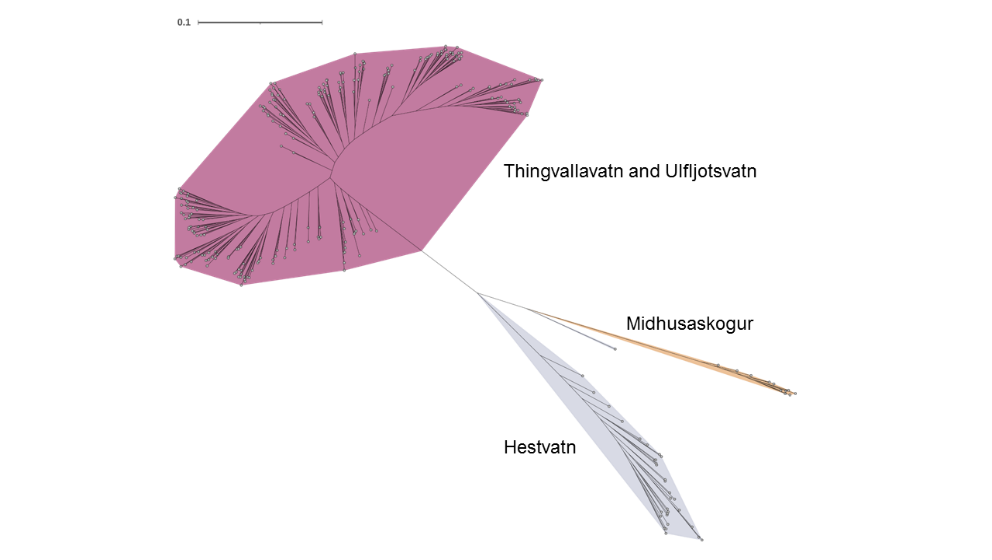
**
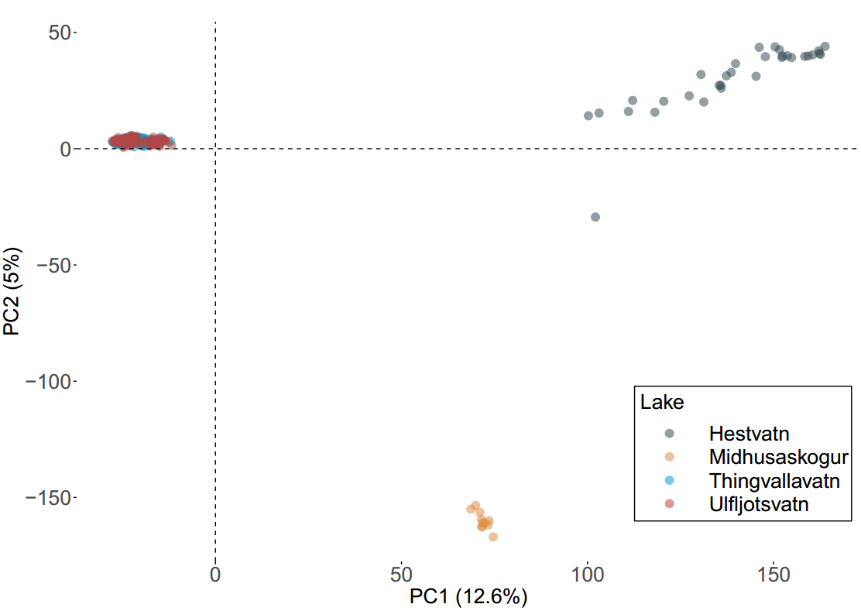
Supplemental Figure S3.** Genetic connectivity and phylogeography of Arctic charr in the Thingvallavatn-Ulfljotsvatn system and reference populations based on 11,160 genome-wide SNPs from the “global” dataset. (a) Principal component analysis showing genetic clustering of individuals along the first and second axis of variation. (b) A maximum-likelihood phylogenetic tree of charr from Thingvallavatn, Ulfljotsvatn, and two reference populations.


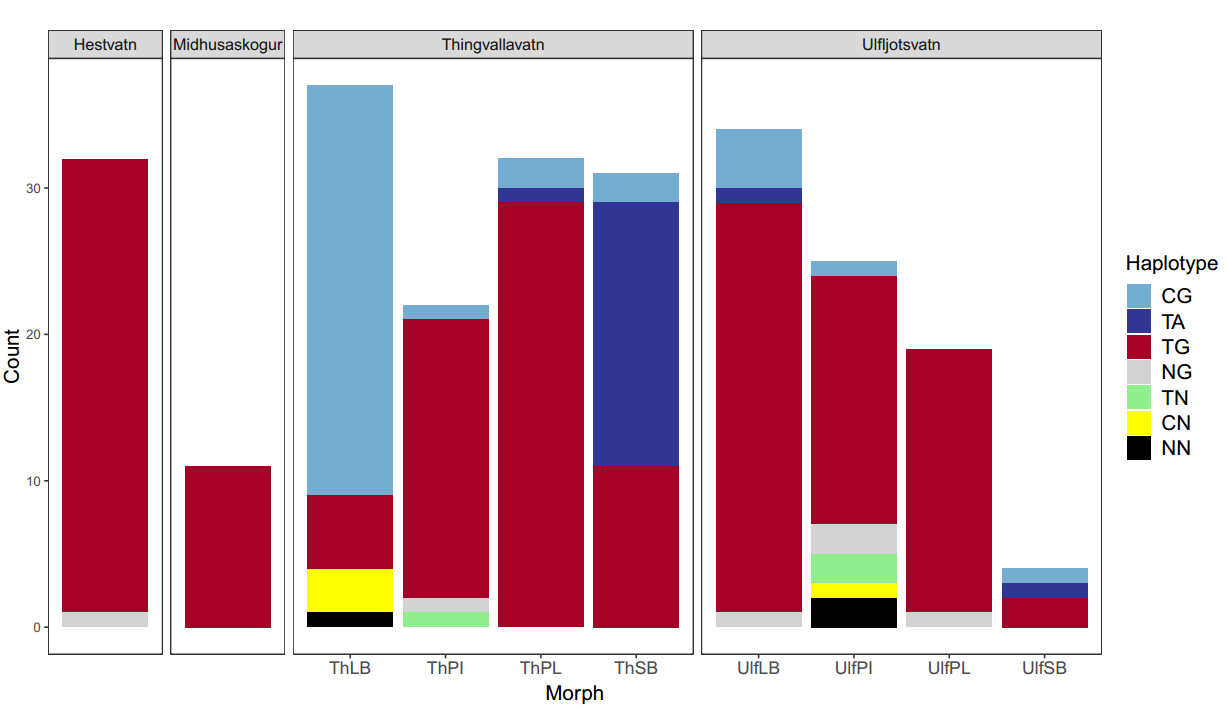


**Supplemental Figure S4.** Stacked bar chart showing the number of individuals carrying unique haplotypes in sympatric morphs in the Thingvallavatn-Ulfljotsvatn system and reference populations. Two markers mt1829 (G>A) and mt3211 (T>C) were genotyped with KASP, resulting in three haplotypes (CG, TA and TG). N in haplotypes indicate that the genotype was not available. Colour in legend denotes the three main haplotypes (and the incompletely genotyped ones).


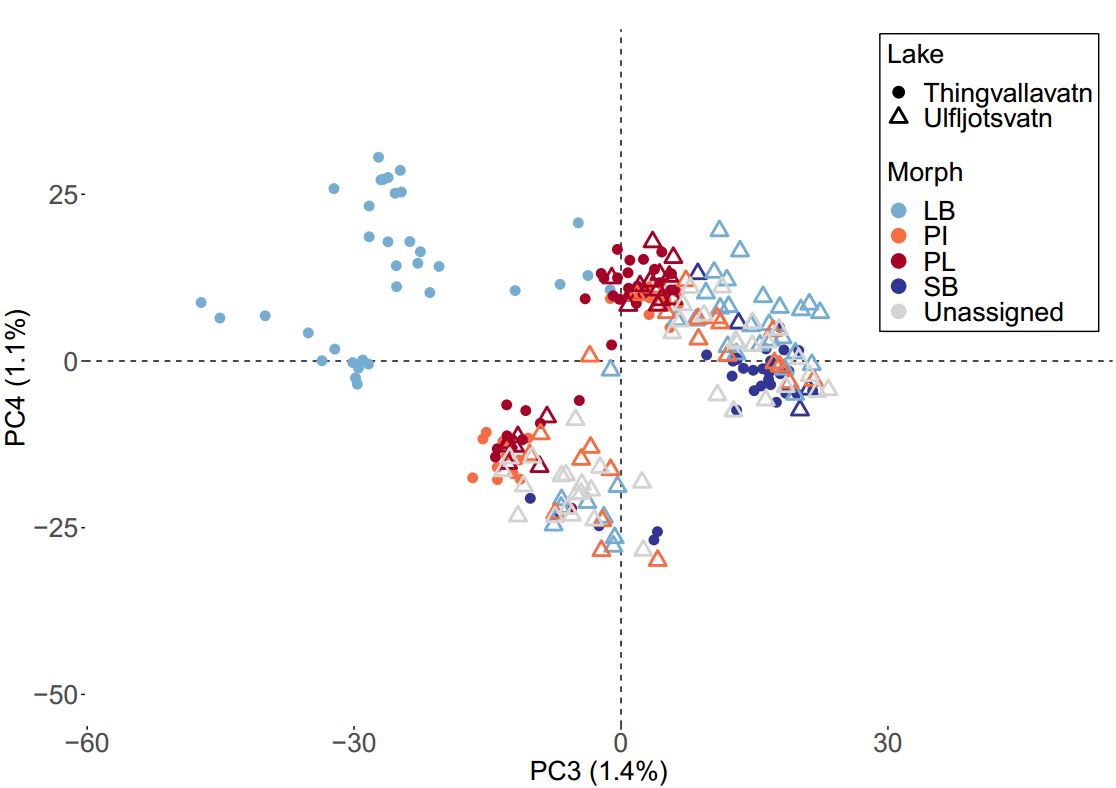


**Supplemental Figure S5.** Principal component analysis showing genetic clustering of Arctic charr in the Thingvallavatn-Ulfljotsvatn system along the third and fourth axis of variation. PC3 separated most of LB-charr from Thingvallavatn from the rest of the morphs, while PC4 separated each morph into two groups (potentially due to sex-related bias, and/or technical reasons, such as library effect ).


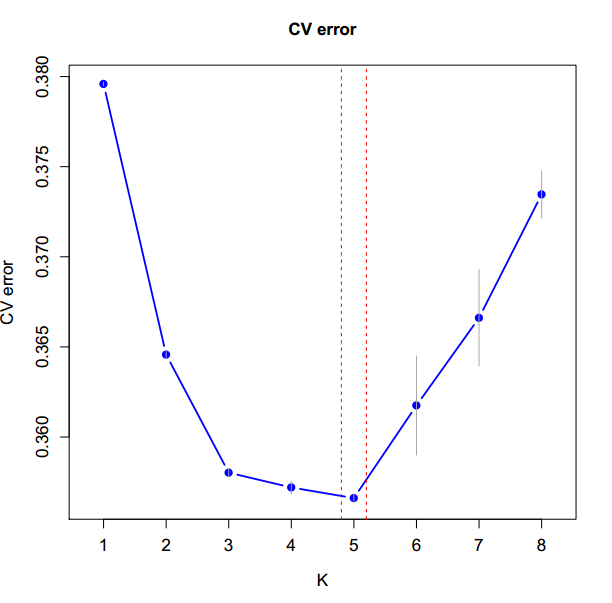

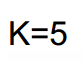

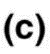

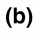

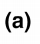

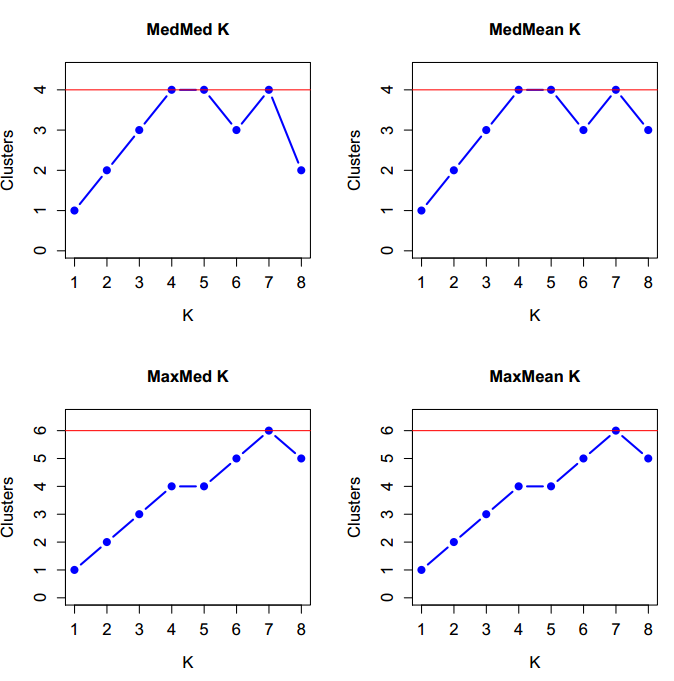

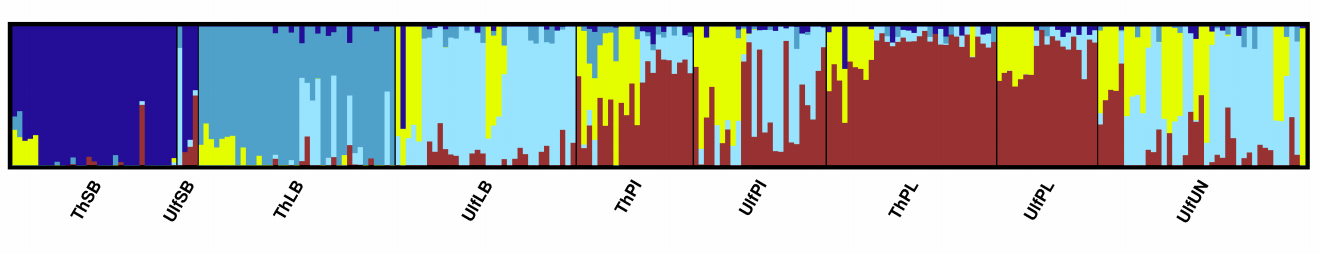


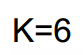

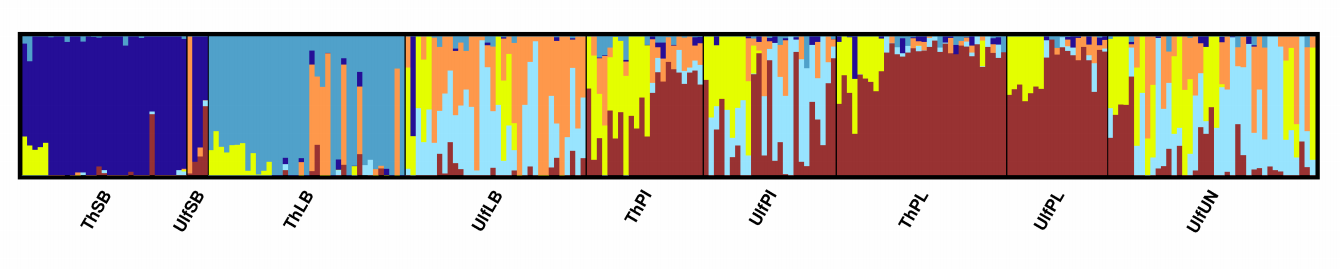


**Supplemental Figure S6.** Information from the Admixture analyses. (a) Cross-validation error of K values ranges from 1 to 8. (b) Estimates of the number of clusters based on MedMedK, MedMeanK, MaxMedK, and MaxMeanK methods. (c) Admixture plots showing the proportion of genetic ancestry for five (K = 5) and six (K = 6) genetic clusters.


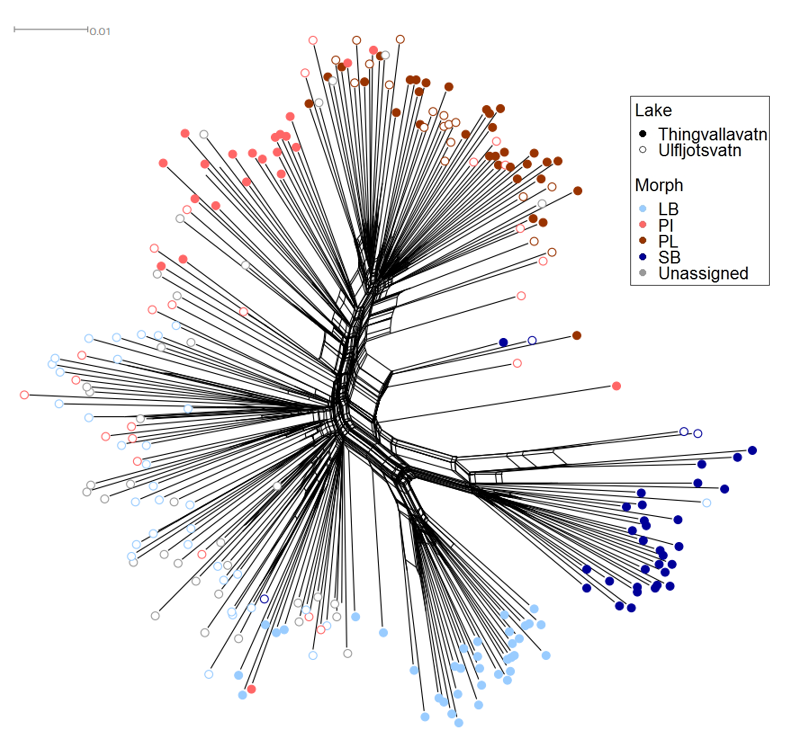


**Supplemental Figure S7.** Neighbor-Net network of Arctic charr in the Thingvallavatn-Ulfljotsvatn system implemented in SplitsTree. Each dot represents an individual. Morph and lake of origin are illustrated by dot colour and fill, see the legend.

**Supplemental Figure S8.** Information from analyses of tree graphs with migration edges inferred by Treemix. (a) A Maximum likelihood tree of all Thingvallavatn charr morphs with one migration edge (left) and the second-order rate of change (Δm) across values of m (right). (b) A Maximum likelihood tree of three abundant charr morphs from Ulfljotsvatn (UlfSB-charr excluded) with one migration edge (left) and the second-order rate of change (Δm) across values of m (right). (c) the second-order rate of change (Δm) across values of m for the maximum likelihood tree of abundant charr morphs (UlfSB-charr excluded) from both Thingvallavatn and Ulfljotsvatn. The optimal number of migration edge is detected by the highest Δm.


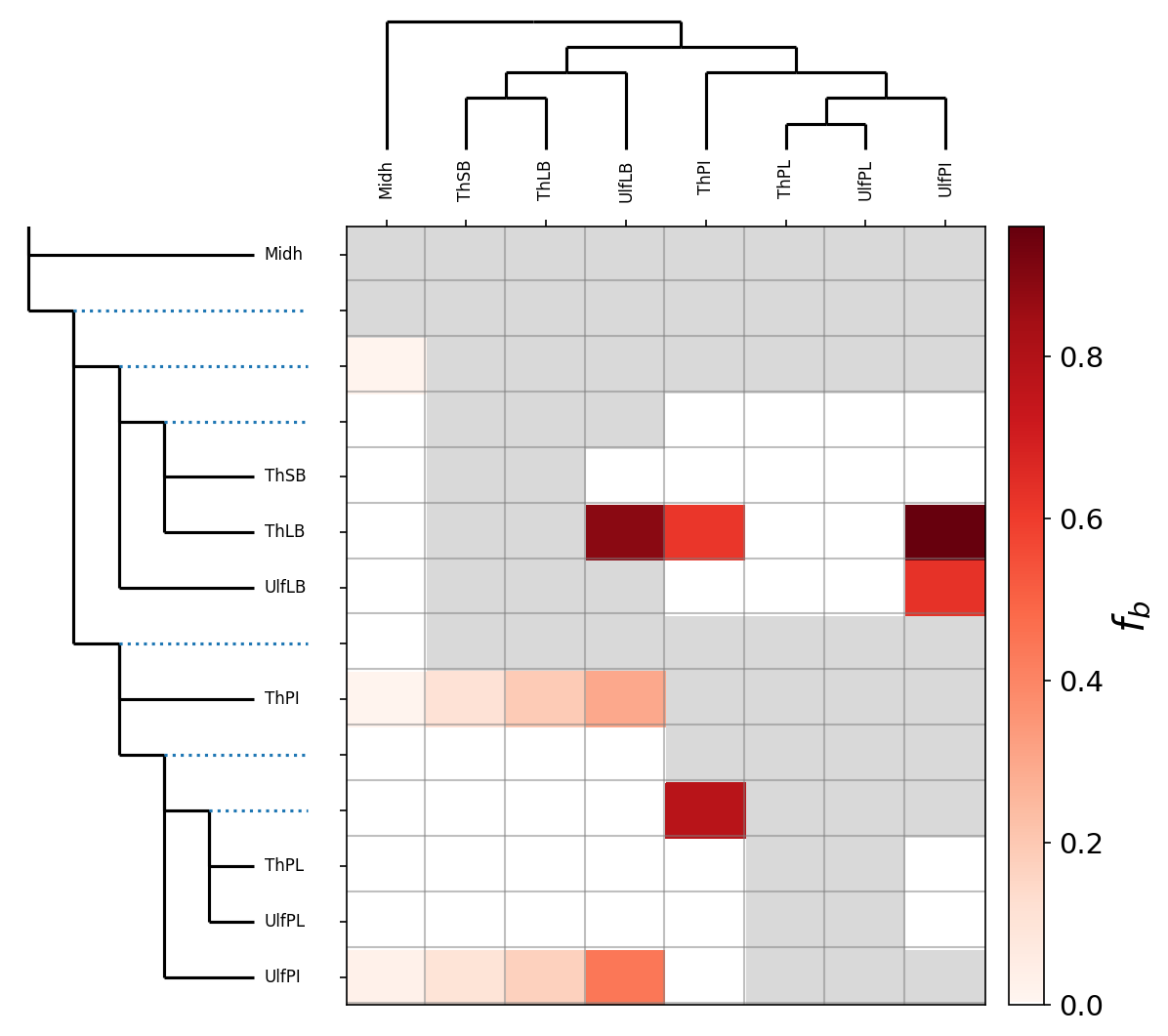


**Supplemental Figure S9.** Results of the f-branch statistic (summary of correlated f_4_-ratio results) for the charr morphs in the Thingvallavatn-Ulfljotsvatn system, including reference populations. Each pairwise f-branch value is represented by a coloured cell in the heatmap according to the accompanying scale bar, where higher values indicate higher levels of introgression. Grey shading denotes comparisons that are not applicable due to phylogeny constraints. The exceptionally strong signal from ThLB to UlfPI in the f4-ratio likely resulted from confounding effects of complex introgression rather than true introgression.
